# Computational evidence for an early, amplified systemic inflammation program in polytrauma patients with severe extremity injuries

**DOI:** 10.1371/journal.pone.0217577

**Published:** 2019-06-04

**Authors:** Khalid Almahmoud, Andrew Abboud, Rami A. Namas, Ruben Zamora, Jason Sperry, Andrew B. Peitzman, Michael S. Truitt, Greg E. Gaski, Todd O. McKinley, Timothy R. Billiar, Yoram Vodovotz

**Affiliations:** 1 Department of Surgery, Division of Trauma & Critical Care Surgery, University of Pittsburgh, Pittsburgh, PA, United States of America; 2 Department of Graduate Medical Education, Department of Surgery, Methodist Dallas Health System, Dallas, TX, United States of America; 3 Center for Inflammation and Regenerative Modeling, McGowan Institute for Regenerative Medicine, University of Pittsburgh, Pittsburgh, PA, United States of America; 4 Department of Orthopedic Surgery, Indiana University School of Medicine, Indianapolis, IN, United States of America; Medical College of Georgia, Augusta, UNITED STATES

## Abstract

Extremity and soft tissue injuries contribute significantly to inflammation and adverse in-hospital outcomes for trauma survivors; accordingly, we examined the complex association between clinical outcomes inflammatory responses in this setting using *in silico* tools. Two stringently propensity-matched, moderately/severely injured (Injury Severity Score > 16) patient sub-cohorts of ~30 patients each were derived retrospectively from a cohort of 472 blunt trauma survivors and segregated based on their degree of extremity injury severity (above or below 3 on the Abbreviated Injury Scale). Serial blood samples were analyzed for 31 plasma inflammatory mediators. In addition to standard statistical analyses, Dynamic Network Analysis (DyNA) and Principal Component Analysis (PCA) were used to model systemic inflammation following trauma. Patients in the severe extremity injury sub-cohort experienced longer intensive care unit length of stay (LOS), total LOS, and days on a mechanical ventilator, with higher Marshall Multiple Organ Dysfunction (MOD) Scores over the first 7 days post-injury as compared to the mild/moderate extremity injury sub-cohort. The higher severity cohort had statistically significant elevated lactate, base deficit, and creatine phosphokinase on first blood draw, along with significant changes in multiple circulating inflammatory mediators. DyNA pointed to a sustained role for type 17 immunity in both sub-cohorts, along with IFN-γ in the severe extremity injury group. DyNA network complexity increased over 7 days post-injury in the severe injury group, while generally decreasing over this same time period in the mild/moderate injury group. PCA suggested a more robust activation of multiple pathways in the severe extremity injury group as compared to the mild/moderate injury group. These studies thus point to the possibility of self-sustaining inflammation following severe extremity injury vs. resolving inflammation following less severe extremity injury.

## Introduction

Trauma is the leading cause of death for adults under the age of 45 and incurs substantial disability in term of long-term morbidity, higher need for rehabilitation service, as well as greater financial costs [1, 2]. Patient outcomes following trauma are influenced by numerous factors including age [3, 4], gender [5, 6], extent of the injury [7, 8], as well as patient-to-patient variability in inflammatory and pathophysiologic responses [7, 9]. While the progression of post-trauma inflammation is complex, many consider the best predictors of outcomes to be the severity and patterns of the injury itself [7]. This core consideration has driven the development and refinement of multiple trauma scoring systems over the last few decades, among which the Injury Severity Score (ISS) remains the most commonly used [10, 11]. However, it is becoming increasingly recognized that response to injury, primarily mediated by the immune system, affects both acute and longer-term outcomes after injury [12–15].

Extremity and soft tissue injury are known to be significant contributors to morbid clinical outcomes and poor clinical trajectories for trauma patients [16–18]. As such, the severity of extremity injuries comprises one of the six parameters in calculating the ISS [19]. Several studies have shown that early stabilization of fractures and appropriate management of soft tissue injury decreases short-term complications, improves long-term function, and decreases overall mortality rate [20, 21]. However, these studies are contrasted by other studies in which early aggressive fracture interventions in vulnerable patients (moderate/severe chest injury, acidosis, and hemodynamic instability) worsened acute outcomes and actually led to some cases of death resulting from an exaggerated immunologic response [22, 23] [24]. Taken together, these disparate clinical courses in patients with fractures highlight both the complexity and potency of the immune response to bone injury. Accordingly, understanding how fractures incite, propagate, and perturb the trauma inflammatory response is critical to optimize trauma patient care.

Bone fractures induce the activation of pro-inflammatory as well as anti-inflammatory components of the immune system [18]. This activates the neuroendocrine system, while local tissue destruction and accumulation of toxic byproducts of metabolic respiration leads to release of inflammatory mediators. Extensive tissue injury may result in spillover of these mediators into the peripheral bloodstream, which further sustains and augments a pro-inflammatory response [18, 25]. Furthermore, the development of organ dysfunction has been linked to an uncontrolled immune response, which can lead to organ failure, sepsis, and death [14, 26]. For over two decades, reductionist approaches have attempted to quantify correspondence of individual or small groups of trauma-affected immune mediators with favorable or adverse outcomes [14, 25, 27–35], but there is an increasing focus on addressing the complexity of trauma-induced inflammation and immune dysregulation via computational modeling [12].

In this study, we hypothesized that patients with severe extremity injuries would have a fundamentally distinct temporal and spatial immune response compared to patients with less severe injury. Accordingly, from a large and diverse cohort of blunt trauma survivors, we studied severely injured trauma patients with extremity injury and an ISS> 16 [36]. Given the confounding impact of age [37–41], gender [6], and injury severity [7, 42], we derived stringently-matched sub-cohorts of severe extremity/soft tissue injury (AIS≥ 3), and mild/moderate extremity injury (AIS< 3) patients that still reflected the primary demographic and injury characteristics of the original large cohort. Our results suggest that severe extremity/soft tissue injury can drive a differential inflammation program associated with self-sustaining inflammation and worse clinical outcomes, as compared to mild/moderate soft tissue injury which is instead associated with a core network of lymphoid inflammatory mediators and self-resolving inflammation.

## Materials and methods

### Patient enrollment, sampling, and clinical data collection

All human sampling was done following approval by the University of Pittsburgh Institutional Review Board, and written informed consent was obtained from each patient or next of kin as per Institutional Review Board regulations. Patients eligible for enrollment in the study were at least 18 years of age, admitted to the intensive care unit (ICU) after being resuscitated, and per treating physician, were expected to live more than 24 h. Reasons for ineligibility were isolated head injury, pregnancy, and penetrating trauma. Laboratory results and other basic demographic data were recorded in the database via direct interface with electronic medical record. Three plasma samples, starting with the initial blood draw upon arrival, were assayed within the first 24 hours following trauma and then from days 1 to 7 post-injury. The blood samples were centrifuged, and plasma aliquots were stored in cryoprecipitate tubes at -80°C for subsequent analysis of inflammatory mediators. Clinical data, including Injury Severity Score (ISS), Abbreviated Injury Scale (AIS), ICU length of stay (LOS), hospital LOS, days on mechanical ventilator, Marshall Multiple Organ Dysfunction (MOD) score, heart rate, blood pressure, Shock Index (S), pH, lactic acid, base deficit (BD), Creatine Phosphokinase (CPK), hematological profile, blood transfusion needs, and surgical interventions were collected from hospital inpatient electronic trauma registry database. ISS [10, 19] and AIS [43, 44] were calculated for each patient by a single trauma surgeon after attending radiology evaluations were finalized. The Marshall MODScore was calculated as index of organ dysfunction, according to Marshall *et al* [45].

### Study design and selection criteria

This was a retrospective case control study, the salient characteristics of which were described recently [46, 47]. Clinical data from 472 blunt trauma survivors (330 males and 142 females, age 48.4 ± 0.9, ISS 19.6 ± 0.5) who were admitted to the Emergency Department of UPMC Presbyterian Hospital, a level 1 trauma center, between January 2004 and January 2012, were examined for the presence of extremity/soft tissue fracture in moderately/severely injured polytrauma patients (ISS > 16). This resulted in a cohort of 198 patients (prevalence = 42%) with extremity/soft tissue injury. This cohort consisted of 128 males and 70 females, mean age 43 ± 1.3 and mean ISS of 27.9 ± 0.7. Patients in this cohort were classified into two sub-cohorts according to the severity of extremity/soft tissue injury (AIS-5): mild/moderate (AIS< 3; n = 134), and severe (AIS≥ 3; n = 64).

In an attempt to reduce the impact of any confounding factors present in the general cohort, we utilized more stringent filtering criteria, as our group has published recently [7, 47–49]. First, we excluded patients with known chronic co-morbidities (hypertension and diabetes), on chronic medications (immune suppressant, steroid), severe head injury (AIS-1≥ 3), and documented alcohol intoxication upon presentation to the emergency department from the severe extremity injury patients (n = 58). We then excluded patients for whom fewer than three blood samples in the first 24 h post-injury were available (n = 78). Finally, we matched these severe (AIS≥ 3) extremity injured trauma patients (n = 32) to similarly matched mild/moderate (AIS< 3) extremity injured trauma patients (n = 30) according to age distribution, gender ratio, ISS, and similar mechanism of injury (i.e. Motor Vehicle Accident; MVA).

### Analysis of inflammation biomarkers

Blood samples were collected into citrated tubes via central venous or arterial catheters within 24 h of admission and daily up to 7 days post-injury. The blood samples were centrifuged, and plasma aliquots were stored in cryoprecipitate tubes at -80°C for subsequent analysis of inflammatory mediators. The human inflammatory MILLIPLEX MAP Human Cytokine/Chemokine Panel-Premixed 24-Plex (Millipore Corporation, Billerica, MA) and a Luminex 100 IS apparatus (Luminex, Austin, TX) were used to measure plasma levels of interleukin (IL)-1β, IL-1 receptor antagonist (IL-1RA), IL-2, soluble IL-2 receptor-α (sIL-2Rα), IL-4, IL-5, IL-6, IL-7, IL-8 (CCL8), IL-10, IL-13, IL-15, IL-17A, interferon (IFN)-γ, IFN-γ inducible protein (IP)-10 (CXCL10), monokine induced by gamma interferon (MIG; CXCL9), macrophage inflammatory protein (MIP)-1α (CCL3), MIP-1β (CCL4), monocyte chemotactic protein (MCP)-1 (CCL2), granulocyte-macrophage colony stimulating factor (GM-CSF), Eotaxin (CCL11), and tumor necrosis factor alpha (TNF-α). The Human Th17 MILLIPLEX Panel kit (Millipore Corporation, Billerica, MA) was used to measure IL-9, IL-21, IL-22, IL-23, IL-17E/25, and IL-33. sST-2 was measured using an ELISA assay (R&D Systems, Minneapolis, MN, catalog # DST-200). The Luminex system was used in accordance to manufacturer’s instructions. NO_2_^-^/NO_3_^-^ was measured using the nitrate reductase/Griess assay (Cayman Chemical Co., Ann Arbor, MI).

### Statistical analysis

All data were analyzed using SigmaPlot 11 software (Systat Software, Inc., San Jose, CA). Statistical difference between severe and mild/moderate extremity injury groups was determined by either Student’s *t*-Test or Chi-square as appropriate. Group-time interaction of plasma inflammatory mediators’ levels between severe and mild/moderate extremity injury groups was determined by Two-Way Analysis of Variance (ANOVA). To quantify the differences between the statistically significant mediators, we calculated the area under the curve (AUC) using the mean values for each time point, and then calculated severe/mild-moderate extremity injury AUC fold change. *P*<0.05 was considered statistically significant for all analyses.

### Dynamic Network Analysis (DyNA)

The goal of this analysis was to gain insights into the temporal dynamic changes in network connectivity and complexity of the post-traumatic inflammatory response between the AIS< 3 and AIS≥ 3 sub-cohorts. The mathematical formation of this method is essentially to calculate the correlation among variables by which we can examine their dependence. To do so, inflammatory mediator networks were created in adjacent 1-day time periods over the 7 days using MATLAB (The MathWorks, Inc., Natick, MA) as we have done previously [46, 47, 50, 51]. Connections in the network were created if the correlation coefficient between two nodes (inflammatory mediators) was greater or equal to a threshold of 0.7. For the network density calculation, in order to account for network sizes (number of significantly altered nodes) in the adjacent 8–h time periods detailed above, we utilized the following formula:

$$\frac{Total\;number\;of\;edges*Number\;of\;total\;nodes}{maximum\;possible\;edges\;among\;total\;nodes}$$

### Principal Component Analysis

Principal Component Analysis (PCA) [50, 52] was carried out to identify those inflammatory mediators that were the most characteristic of the overall dynamic, multivariate response of a given patient sub-group using MATLAB software (The MathWorks, Inc., Natick, MA). To perform this analysis, the data were first normalized for each inflammatory mediator (i.e. a given value divided by the maximum value for a given inflammatory mediator), so that all mediator levels were converted into the same scale (from 0 to 1). In this way, any artifactual effects on variance due to the different ranges of concentration observed for different cytokines were eliminated. Only sufficient components to capture at least 95% of the variance in the data were considered. From these leading principal components, the coefficient (weight) associated with each inflammatory mediator was multiplied by the eigenvalue associated with that principal component. This product represented the contribution of a given mediator to the variance accounted for in that principal component. The overall score given to each mediator is the sum of its scores in each component, depicted as a stacked bar graph. This gives a measure of a given inflammatory mediator’s contribution to the overall variance of the system. The mediators with the largest scores are those which contributed most to the variance of the process being studied [50, 52].

## Results

### Demographics and clinical outcomes for the overall cohort

Our derivation cohort of 472 blunt trauma survivors has been described extensively in prior publications [46, 47]. The majority of 472 patient cohort were male (330/472; 70%), with a mean age of 48.4 ± 0.9 years and a mean ISS of 19.6 ± 0.5. These patients sustained blunt trauma predominantly in the form of motor vehicle accidents and falls. The mean of ICU LOS was 6.9 ± 0.4 days, the mean hospital LOS was 12.7 ± 0.5 days, and the mean number of days on a mechanical ventilator was 2.9 ± 0.3 days.

We next examined the sub-group of patients with extremity fractures (198 patients). These patients sustained blunt trauma in form of motor vehicle accidents. Males were predominant in our 198-patients cohort with extremity injury (128/198; 65%), with a mean age of 43 ± 1.3 years and a mean ISS of 27.9 ± 0.7. There was no statistical difference in mean age (*P* = 0.7), and ISS (*P* = 0.7) between the two cohorts. The mean of ICU LOS was 9.3 ± 0.6 days, the mean hospital LOS was 15.7 ± 0.7 days, and the mean number of days on a mechanical ventilator was 5.2 ± 0.5 days.

The severe extremity injury group had a statistically higher (*P* = 0.001) requirement for transfusion (23/64 patients [36%]) as compared to the mild/moderate injury group (8/134 patients [6%]). To control for the potential confounding effects of severe hemorrhage, we assessed the Shock Index (heart rate/systolic blood pressure). This analysis indicated no significant differences between the severe extremity injury vs. the mild/moderate injury cohorts (0.91 ± 0.35 vs. 0.82 ± 0.27 [mean ± SEM]; *P* = 0.185 by Mann-Whitney U test).

Moreover, the ICU LOS (*P* = 0.001), hospital LOS (*P* = 0.001), and days on mechanical ventilator (*P*<0.001) were all statistically significantly longer in the severe extremity injury cohort as compared to the mild/moderate extremity injury cohort.

### Overall demographics and clinical outcomes of stringently-matched extremity/soft tissue injury sub-cohorts

To test our hypothesis regarding differential trajectories and networks of systemic inflammation as a function of extremity injury severity, we sought to derive stringently-matched sub-cohorts from our derivation cohort that would be as similar as possible with regard to their basic demographics. As part of this process, we focused on the most common mechanism of injury. Thus, from the above-described overall patient cohort of 472 trauma survivors, 62 patients were selected for this study: a sub-cohort of 30 patients with mild/moderate extremity injury, and a sub-cohort of 32 patients with severe extremity injury (See *Materials and Methods*). In this selection process, we focused on motor vehicle accidents as this was the most common mechanism of injury.

**Fig 1:**
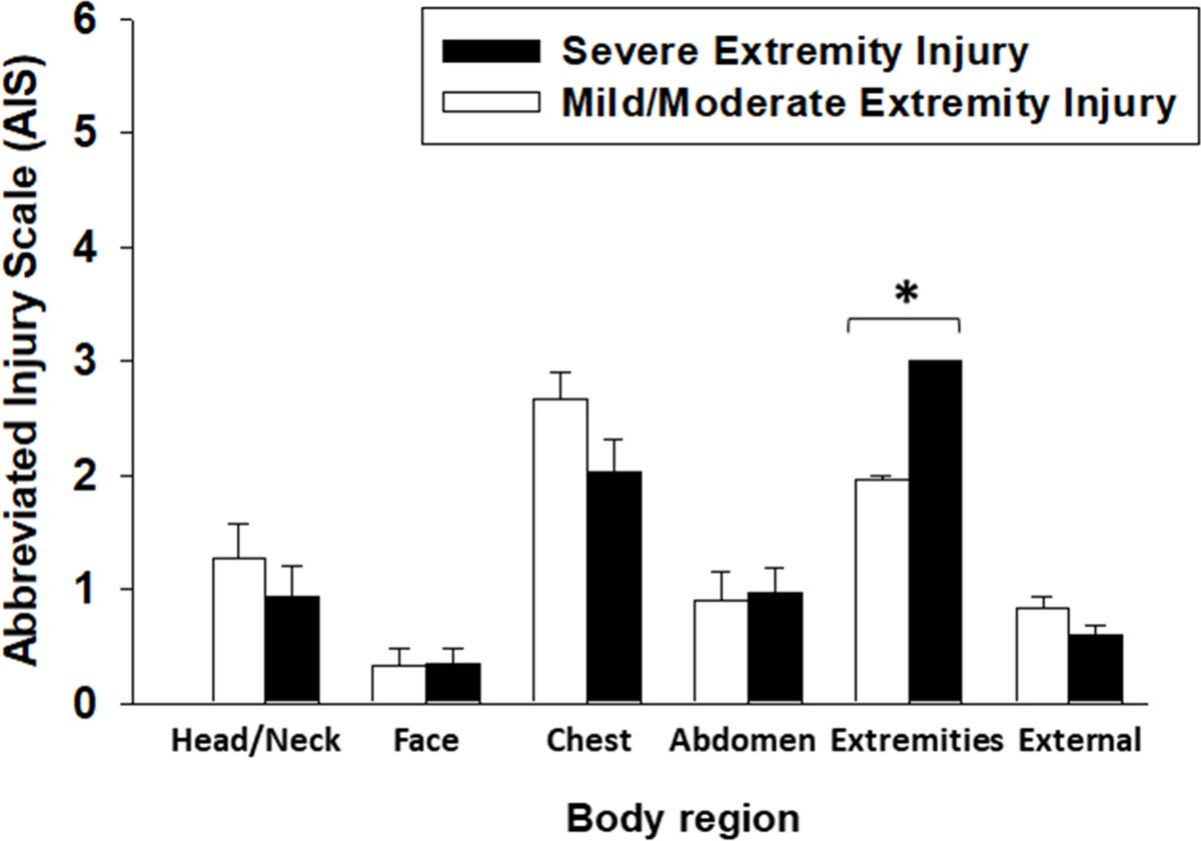
Stringently matched sub-cohorts differ only in the extremities component of the Abbreviated Injury Scale. Trauma patients were recruited following IRB approval and informed consent. The Abbreviated Injury Scale (AIS) score was statistically significantly higher in the extremities regions in the severe extremity injury sub-cohort when compared to a stringently matched mild/moderate extremity injury sub-cohort (*P<0.05 vs. mild injury analyzed by One-Way ANOVA).

Overall, males were predominant in both severe extremity and mild/moderate extremity injury cohorts (18/32 (56%) and 15/30 (50%), respectively), with no statistical difference in mean age (*P* = 0.8) or ISS (*P* = 0.6) between the two cohorts. Statistically significant differences were observed only in the extremities component (AIS-5; *P*<0.001) of the Injury Severity Score of severely-injured sub-cohort when compared to mild/moderate extremity injured sub-cohorts (Fig 1). Moreover, the ICU LOS (*P* = 0.017), hospital LOS (*P* = 0.001), and days on mechanical ventilator (*P*<0.001) were all statistically significantly longer in the severe extremity injury cohort when compared to the mild/moderate cohort (Table 1).

**Table 1:** Demographic data, clinical characteristics and outcome of stringently matched sub-cohorts (Severe extremity injury cohort n = 32, Mild/Moderate extremity injury cohort n = 30). Values are mean ± SEM. Statistical significance set at *P*<0.05 by either Student’s *t*-Test or Chi-square as appropriate.

	Mild/Moderate Extremity Injury n = 30	Severe Extremity Injury n = 32	*P- Value*
***Demographics***			
**Age (years)**	53.2 ± 2.4	52.5 ± 3.1	0.8
**Sex (male: female)**	15:15	18:14	0.9
**Injury severity score (ISS)**	22.4 ± 1.4	21.5 ± 1.5	0.6
***Mechanism of injury***			
**Motor vehicle accidents (MVA), n (%)**	30 (100%)	32 (100%)	0.9
**Open fracture, n (%)**	3 (10.0%)	6 (18.7%)	0.8
**Vascular injury present, n (%)**	2 (6.7%)	3 (9.4%)	0.9
**Vertebral injury present, n (%)**	4 (13.3%)	11 (34.4%)	0.01
***Outcome***			
**Intensive Care Unit length of stay (days)**	5.9 ± 1.3	10.3 ± 1.5	0.02
**Mechanical ventilator (days)**	1.7 ± 0.9	7.2 ± 1.5	<0.001
**Hospital length of stay (days)**	11.4 ± 1.5	18.8 ± 1.9	0.001
***Surgical Interventions***			
**Fracture Fixation, n (%)**	8 (26.8%)	20 (62.5%)	0.01
**Laparotomy, n (%)**	6 (20.0%)	18 (56.3%)	0.01
**None, n (%)**	22 (73.3%)	12 (37.5%)	0.01
***Complete Blood Counts* 0.78**			
**White blood cells**	15.8	16.6	N/A
**Hemoglobin**	12.4	11.8	N/A
**Hematocrit**	36.3	34.8	N/A
**Platelets**	228.0	236.6	N/A
**Neutrophils (%)**	72.9	74.6	N/A
**Lymphocytes (%)**	16.1	15.0	N/A
**Monocytes (%)**	6.4	5.6	N/A
**Eosinophils (%)**	1.1	0.7	N/A
**Basophils (%)**	0.1	0.2	N/A
**Prothrombin time (PT)**	14.8	15.8	N/A
**International Normalization Ratio (INR)**	1.2	1.3	N/A
**Partial Thromboplastin Time (PTT)**	26.0	27.5	N/A
***Complications* 0.25**			
**Transfusion of blood products, n (%)**	3 (10%)	12 (37.5%)	N/A
**Nosocomial infection, n (%)**	8 (26.8%)	14 (43.8%)	N/A
**Pneumonia, n (%)**	2 (6.7%)	8 (25%)	N/A
**Urinary tract infection, n (%)**	3 (10.0%)	5 (15.6%)	N/A
**Bloodstream infection, n (%)**	2 (6.7%)	3 (9.4%)	N/A
**Pseudo-membranous colitis, n (%)**	1 (3.3%)	2 (6.3%)	N/A
**Wound Infection, n (%)**	2 (6.7%)	4 (12.5%)	N/A
**None, n (%)**	22 (73.3%)	18 (56.3%)	N/A
***Disposition* 0.80**			
**Inpatient rehabilitation facility, n (%)**	3 (10.0%)	2 (6.3%)	N/A
**Home, n (%)**	10 (33.3%)	7 (21.9%)	N/A
**Home with service, n (%)**	2 (6.7%)	3 (9.4%)	N/A
**Rehabilitation, n (%)**	3 (10.0%)	3 (9.4%)	N/A
**Skilled nursing facility, n (%)**	12 (40.0%)	17 (53.1%)	N/A

As in the parent extremity injury cohorts, the severe extremity injury sub-cohort had a similar and significantly higher (*P* = 0.026) incidence of transfusion (12/32 patients; 37.5%) vs. the mild/moderate extremity injury patients (3/30; 10%). Again, assessment of the Shock Index (heart rate/systolic blood pressure) indicated no significant differences between the severe extremity injury sub-cohort (1.02 ± 0.173 [mean ± SEM]) vs. the mild/moderate injury sub-cohort (0.82 ± 0.044) (*P* = 0.95 by Mann-Whitney U test).

### Clinical biochemistry parameters are altered significantly as a function of extremity/soft tissue injury severity

**Fig 2:**
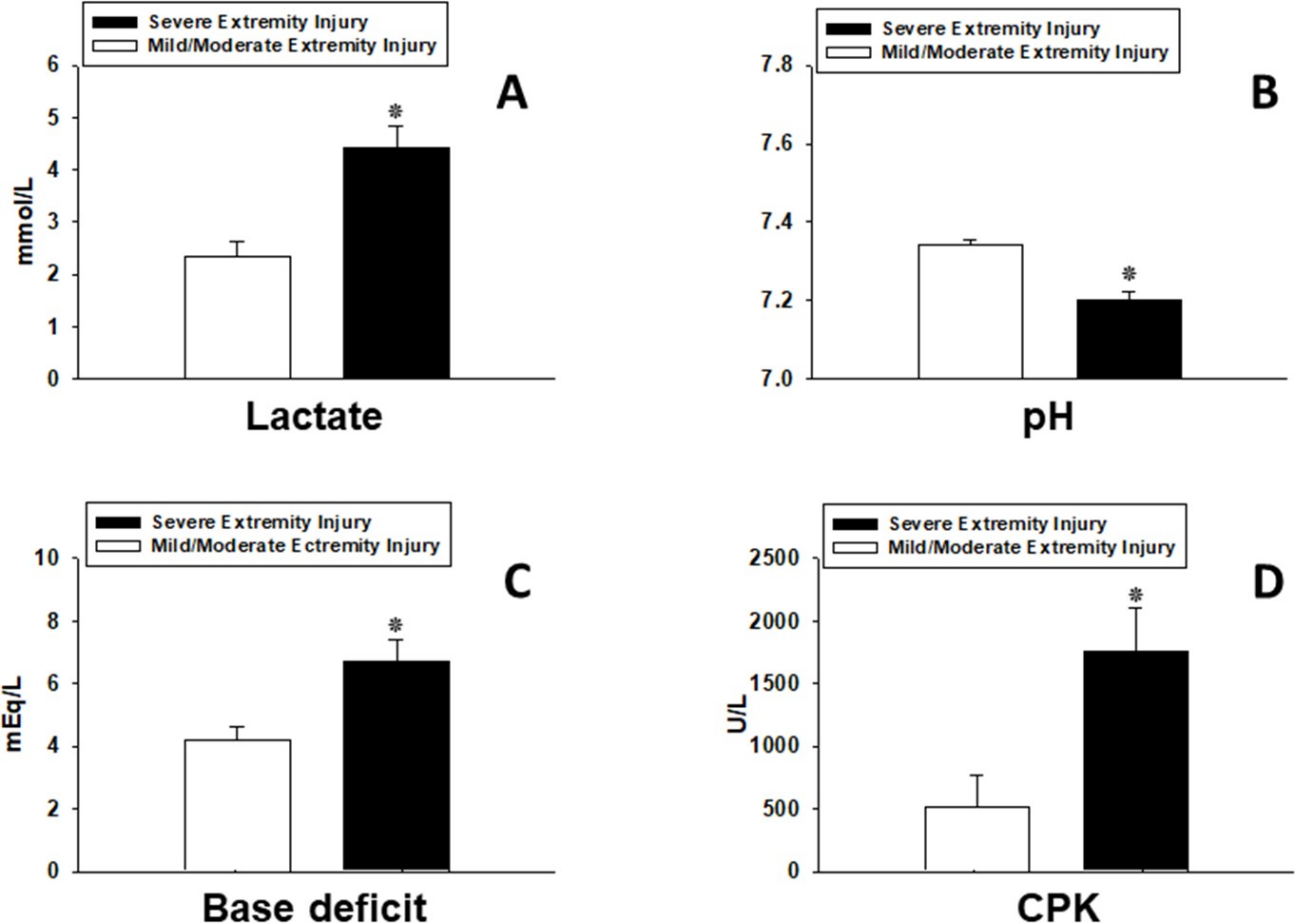
Clinical biochemistry parameters are significantly altered as a function of extremity/soft tissue injury severity in stringently matched sub-cohorts. (**A**) Plasma lactate levels were statistically significant higher in the severe extremity injury sub-cohort when compared to a stringently matched mild/moderate extremity injury sub-cohort over the first 24 h post injury. (**B**) pH was statistically significant lower in the severe extremity injury sub-cohort when compared to a stringently matched mild/moderate extremity injury sub-cohort over the first 24 h post injury. (**C**) Base deficit (BD) was statistically significantly higher in the severe extremity injury sub-cohort when compared to a stringently matched mild/moderate extremity injury sub-cohort over the first 24 h post-injury. (**D**) Creatine Phosphokinase (CPK) was statistically significantly higher in the severe extremity injury sub-cohort when compared to a stringently matched mild/moderate extremity injury sub-cohort over the first 24 h post-injury.

Abnormalities in admission biochemical parameters (pH, Lactate, CPK, hematocrit, etc.) in trauma patients are associated with higher mortality and morbidity and were used to define the “golden hour” for surgical intervention [53, 54]. In this context, we evaluated lactate, base deficit, pH, Creatine Phosphokinase (CPK), hemoglobin (Hbg), hematocrit (Hct), platelet counts, and white blood cell counts (WBC) in our stringently-matched sub-cohorts. This analysis showed that lactate (4.4 ± 0.4 vs. 2.3 ± 0.3; *P*<0.001; Fig 2A), pH (7.2 ± 0.02 vs. 7.3 ± 0.01; *P*<0.001; Fig 2B), base deficit (BD; 6.7 ± 0.7 vs. 4.2 ± 0.5; *P* = 0.021; Fig 2C), and CPK (1756 ± 345 vs. 509 ± 255; *P* = 0.03; Fig 2D) assessed upon admission were significantly different in patients with severe extremity injuries when compared to patients with mild/moderate extremity injuries.

### Greater requirement for surgical intervention as a function of extremity/soft tissue injury severity

As expected, patients with severe extremity/soft tissue injury were in greater need of surgical interventions (n = 20, 63%) in comparison to the mildly/moderately injured group (n = 8, 27%). Moreover, 6/30 (20%) mild/moderate extremity injury patients, and 18/32 (56%) severe extremity injury patients underwent exploratory laparotomy to identify and control a source of bleeding. Furthermore, 8/30 (27%) patients in the mild/moderate extremity injury sub-cohort, and 20/32 (63%) in the severe extremity injury sub-cohort had either simple or compound fractures that required additional orthopedic surgical procedures. Finally, 22/30 (73%) of the mild/moderate injury patients, and 12/32 (37%) of the severe injury patients did not require any surgical intervention through their clinical course (*P* = 0.01; Table 1).

### Greater severity of multiple organ dysfunction is a function of extremity/soft tissue injury severity

**Fig 3:**
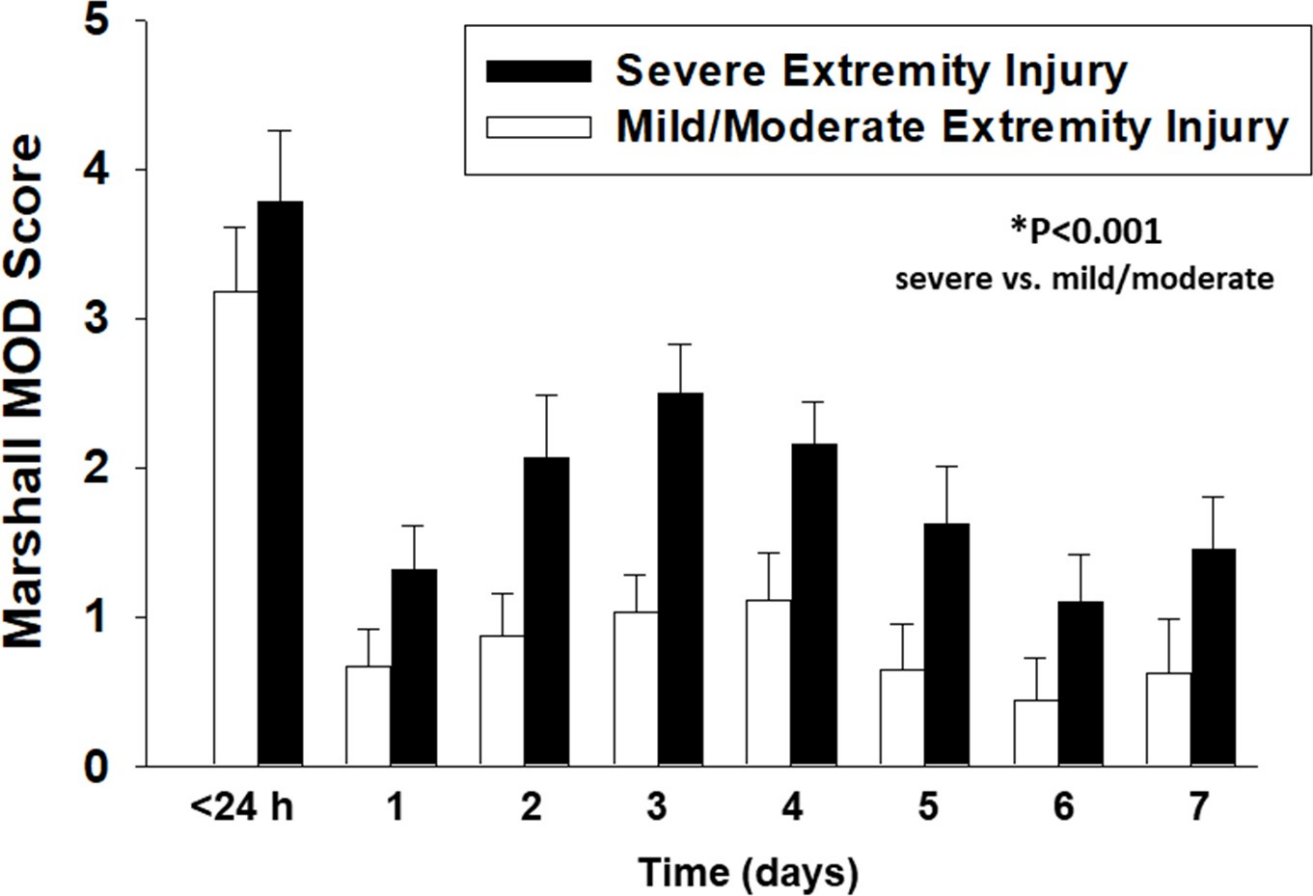
Greater severity of multiple organ dysfunction as a function of extremity/soft tissue injury severity over 7 days. Daily Marshall MODScore analysis of organ failure suggests that the severe extremity injury sub-cohort had higher degree of MOD from day 2 up to day 7 post-injury when compared to a stringently matched mild/moderate extremity injury sub-cohort. *P<0.05 by Two-Way ANOVA.

The two sub-cohorts varied in their degree of MOD, as indicated by the Marshall MODScore, a well-validated index of dysfunction in multiple organ systems [45, 55]. Marshall MODScores were calculated for each time point at which inflammation biomarkers were assessed. This analysis suggested that patients with severe extremity injury had a statistically significantly higher degree of organ dysfunction (*P*<0.001)—when compared to the patients with mild/moderate extremity injuries (Fig 3). Notably, significant differences were observed in the neurological, respiratory, and renal component scores of the Marshall MODScore when comparing mild/moderate vs. severe extremity injury sub-cohorts (S2 Fig).

### Different trajectories of systemic inflammation as a function of extremity injury severity

**Fig 4:**
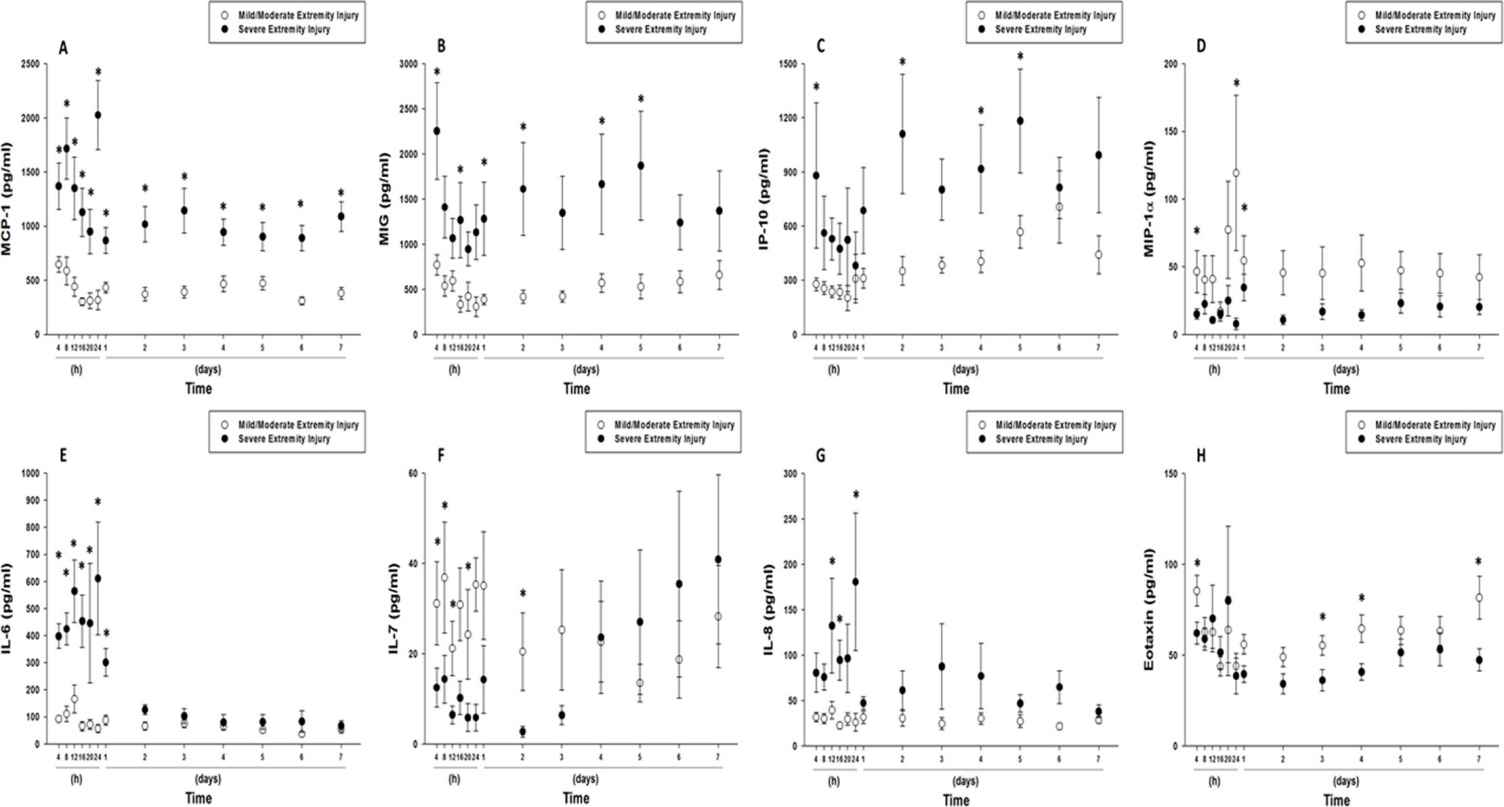
Time course analysis of inflammation biomarkers in the mild/moderate, and severe extremity injury sub-cohorts from time of injury up to 7 days. Trauma patients were recruited following IRB approval and informed consent. Plasma was obtained at multiple time points and analyzed for the presence of 27 inflammatory mediators in highly-matched sub-cohorts of patients with severe vs. mild/moderate extremity injury described in *Materials and Methods*. Mean circulating levels of inflammatory mediators in the mild/moderate extremity injury (n = 30), and severe extremity injury (n = 32) sub-cohorts. (**A**) Time course of MCP-1/CCL2. (**B**) Time course of MIG/CXCL9. (**C**) Time course of IP1-0/CXCL10. (**D**) Time course of MIP-1α (CCL3). (**E**) Time course of IL-6. (**F**) Time course of IL-7. (**G**) Time course of IL-8. (**H**) Time course of Eotaxin (CCL11). The indicated inflammatory mediators were assessed in serial plasma samples obtained at the indicated time points. Values are mean ± SEM. *P<0.05 by Two-Way ANOVA.

Extensive time course analysis of circulating inflammation biomarkers over 7 days showed that circulating levels of IL-6 (*P*<0.001), IL-8 (*P*<0.001), IP-10/CXCL10 (*P*<0.001), MIG/CXCL9 (*P*<0.001), and MCP-1/CCL2 (*P*<0.001) were significantly higher, and IL-7 (*P* = 0.017), macrophage inflammatory protein (MIP)-1α (CCL3) (*P*<0.001)–and Eotaxin (CCL11) (*P* = 0.006) were significantly lower–in patients with severe extremity/soft tissue injuries when compared to patients with mild/moderate extremity injuries (Fig 4 and S1 Table). An analysis of area under the curve (AUC), in which circulating inflammatory mediators were ranked according to the fold change (severe/ mild-moderate extremity injury patients) is shown in Table 2.

**Table 2:** Area under the curve (AUC) analysis for the statistically significantly different inflammatory mediators (by Two-Way ANOVA) between the stringently matched sub-cohorts of severe and mild/moderate extremity injured patients during the 7 days’ time course.

Inflammatory Mediators (Time of injury– 7days)	Mild/Moderate Extremity Injury	Severe Extremity Injury	Fold change	*P*-value
**IL-6**	927.7298	3506.819	3.8	**<0.001**
**IL-8**	342.9599	1023.372	3	**<0.001**
**MCP-1**	4919.777	14168.47	2.9	**<0.001**
**MIG**	5811.091	16649.75	2.8	**<0.001**
**IP-10**	4324.87	8919.756	2.1	**<0.001**
**Eotaxin**	712.2358	608.8193	0.9	**0.006**
**IL-7**	313.8759	178.842	0.6	**0.017**
**MIP-1α**	629.5522	219.0117	0.35	**<0.001**

### Differential *in silico*-defined networks of systemic inflammation as a function of extremity injury severity

**Fig 5:**
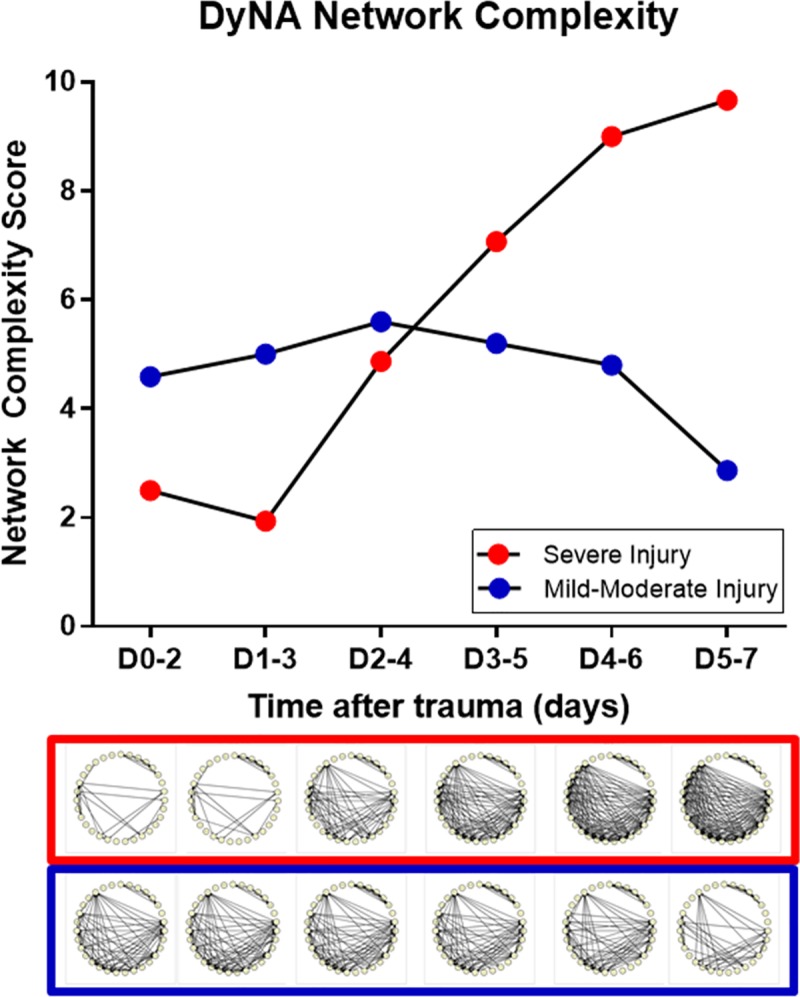
DyNA network complexity identifies inverse inflammation trajectories between trauma patients severe extremity injury vs. a stringently matched sub-cohort with mild/moderate extremity injury. Trauma patients were recruited following IRB approval and informed consent. Plasma was obtained at multiple time points and analyzed for the presence of 31 inflammatory mediators in highly-matched sub-cohorts of patients with severe vs. mild/moderate extremity injury, followed by Dynamic Network Analysis (DyNA) as described in the *Materials and Methods*. The time-evolution of networks in severe (framed in red) vs. mild-moderate (framed in blue) extremity injury is displayed. *In silico* inference of inflammatory network complexity suggests a bifurcation in network progression as time progressed up to 7 days post-injury: the mild/moderate injury group reached inflammation resolution and suppression over 7 days, whereas the severe injury group multiplied in complexity over 7 days, ultimately reaching a higher network complexity than did the mild/moderate group at the onset of inflammation.

Based on these findings, we next hypothesized that the differences in the systemic inflammatory response between the mild/moderate and severe extremity injury cohorts could be explained by differential expression of dynamic networks. As such, we sought to segregate the inflammatory responses in these cohorts in a granular fashion to capture time evolution of networks of systemic inflammation using DyNA. Fig 5 shows the DyNA results for the severe and mild/moderate injury groups over five different time periods from time of traumatic insult up to day 7 post injury. This analysis suggested that patients with an AIS≥3 initially exhibited a lower degree of network connectivity, whereas the mild/moderate group initially exhibited a drastically higher level of network connectivity at up to day 3. Notably, as time progressed up to 7 days post-injury, the levels of inflammatory connectivity in each of the two groups diverged inversely. The dynamic networks in the mild/moderate injury group suggested inflammation resolution and suppression over 7 days, whereas the severe injury group exhibited amplified complexity over 7 days, ultimately reaching a higher network complexity than did the mild/moderate group at the onset of inflammation. Notably, there appears to be a marked peak in inflammatory connectivity around days 2–4, when both sub-cohorts exhibit highly connected inflammatory networks, yet thereafter follow differential trajectories of inflammation and connectivity.

**Fig 6:**
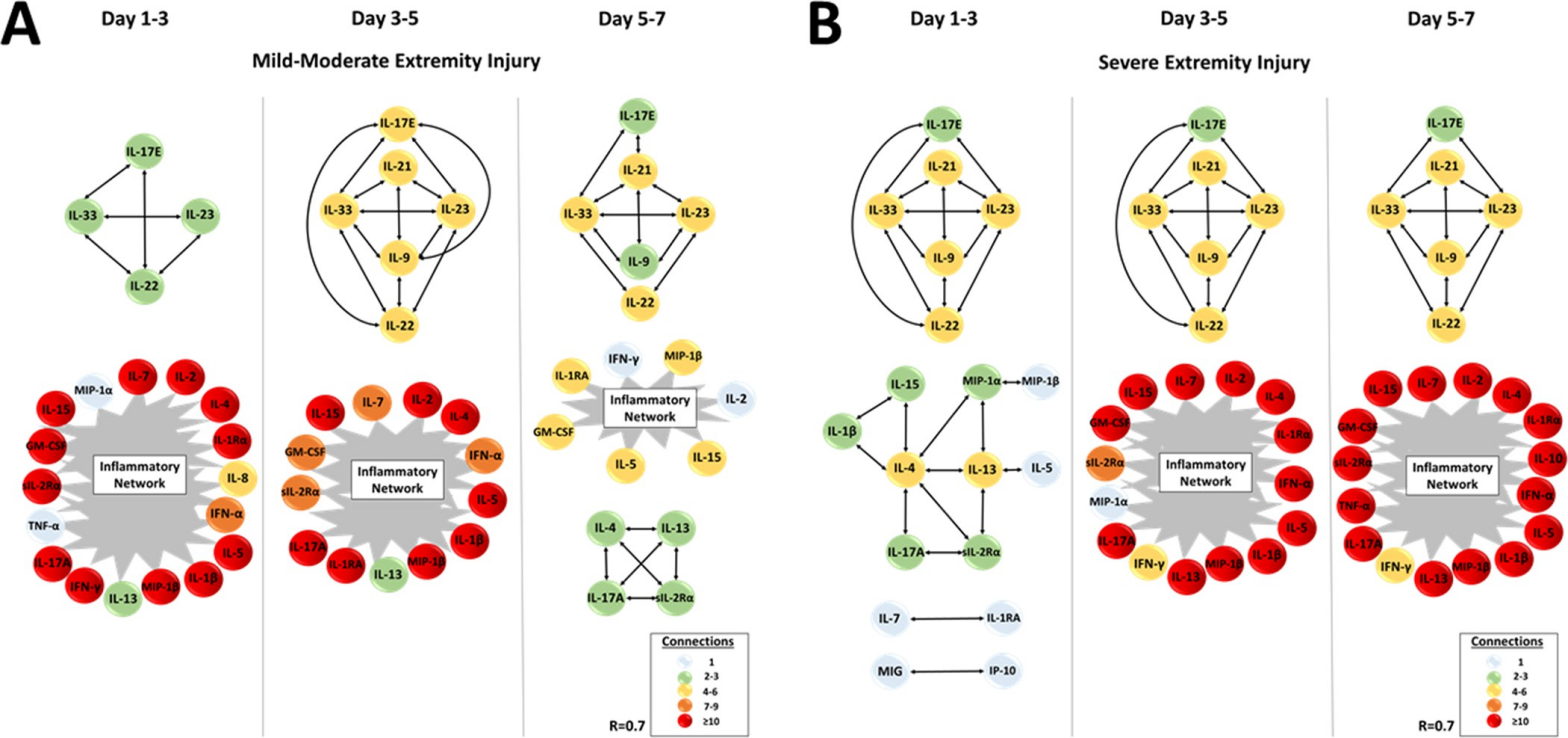
Differential dynamic inflammatory networks identified after 5 days in trauma patients with severe extremity injury vs. a stringently matched sub-cohort of patients with mild/moderate extremity injury. Trauma patients were recruited following IRB approval and informed consent. Plasma was obtained at multiple time points and analyzed for the presence of 31 inflammatory mediators in highly-matched sub-cohorts of patients with severe vs. mild/moderate extremity injury, followed by Dynamic Network Analysis (DyNA) as described in the *Materials and Methods*. **(A)** Inferred dynamic networks in the mild/moderate group suggested a characteristic, lymphoid-predominant, core inflammatory network of sIL-2Rα, IL-4, IL-13, and IL-17A which contains features of a similar, lymphoid-predominant, core inflammatory network associated with survival in blunt trauma patients up to 7 days post-injury [46]. **(B)** The severe extremity injury group was characterized by both innate and lymphoid mediators which evolved into increasingly complex networks of 7 day, with the emergence of the pro-inflammatory TNF-α and anti-inflammatory IL-10 at Day 5–7. The prototypical Th1 cytokine IFN-γ did not appear in the inflammation networks of the mild/moderate sub-cohort until days 5–7, whereas it was increasingly connected in the inflammation networks of the severe sub-cohort over 7 days. All original DyNA outputs are included in S1 Fig.

A more detailed analysis of the specific inflammatory mediators involved within the networks revealed important patterns of inflammation which segregated the two patient sub-cohorts. Strikingly, DyNA inference highlighted a characteristic, lymphoid-predominant, core inflammatory network of sIL-2Rα, IL-4, IL-13, and IL-17A (Fig 6A), with similar features (Treg, Th2, and Th17) to those associated with survival in blunt trauma patients [46]. DyNA also revealed a relatively sustained sub-network in both sub-cohorts that included IL-9, IL-17E, IL-21, IL-22, IL-23, and IL-33. As the number of connections within the mild/moderate extremity injury group decreased in a stepwise fashion, many of the early innate inflammatory mediators disappeared in concert with the appearance of lymphoid and reparative networks. In contrast, the severe extremity injury group displayed an increasingly complex interconnection of innate and lymphoid mediators from day 1 to day 7 (Fig 6B). A host of inflammatory mediators in the severe injury group were highly interconnected, with the emergence of the pro-inflammatory TNF-α and anti-inflammatory IL-10 at day 5–7 (Fig 6B). A quantitative analysis of connectivity shows a 38.3% increase in the number of mediators connected in the severe injury group vs. the mild/moderate group (S2 Table). Interestingly, the hallmark pro-inflammatory cytokine IL-6, which was elevated significantly in the severe extremity injury patients (Fig 4E), was not connected to other mediators in either DyNA output.

### Principal Component Analysis suggests a more robust inflammatory response and differential role for type 2 immune responses following severe extremity fracture

**Fig 7:**
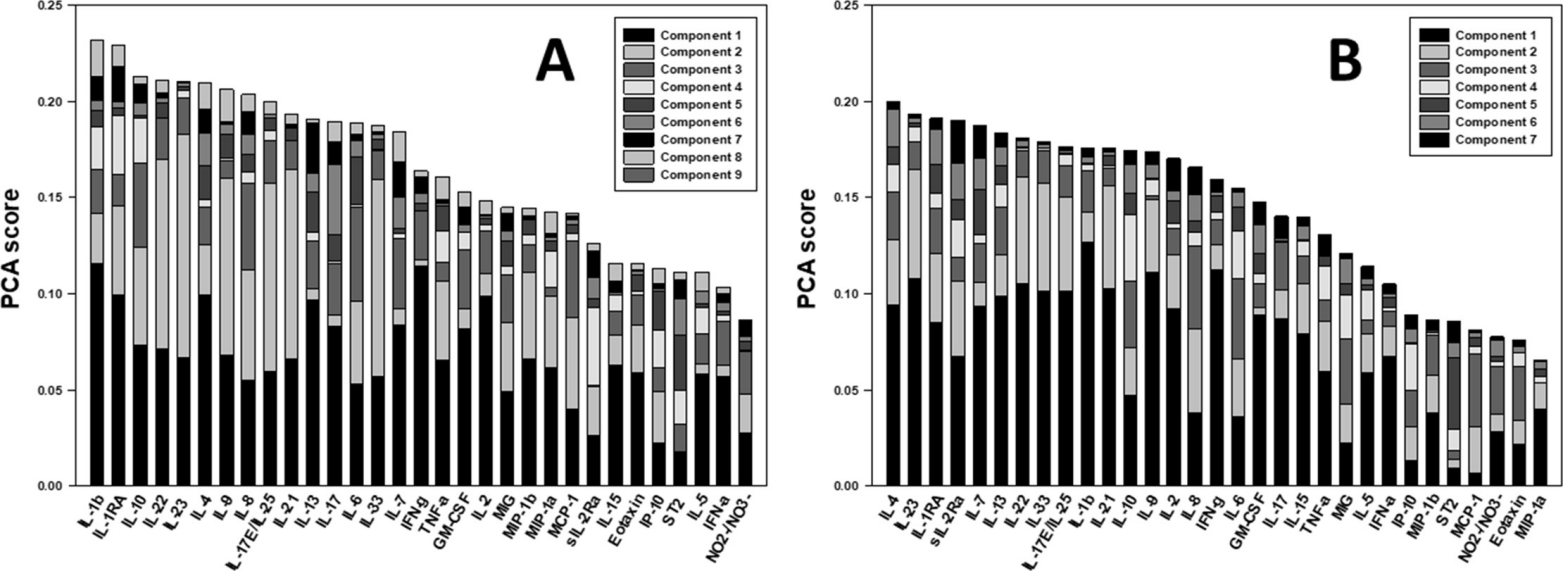
Principal component analysis suggests a differential role for type 2 immune responses in the circulating inflammatory response to extremity injury. Trauma patients were recruited following IRB approval and informed consent. Principal component analysis was carried out using the data from stringently matched sub-cohorts of patients with severe vs. mild/moderate extremity injury as described in the *Materials and Methods*. Both sub-cohorts shared 4 out of the 5 leading principal components: IL-1β, IL-7, IL-13, and IFN-γ, exhibiting a conserved, core inflammatory signature of extremity fracture and/or polytrauma **(A)** PCA of the severe injury group exhibited more components than that of the mild/moderate injury group (9 vs. 7, respectively), as well as having a slightly greater overall magnitude (~0.23 vs. 0.20, respectively). The PCA from day 0–7 identified IL-1β and its antagonist IL-1RA as leading principal mediators in the severe extremity injury group but not in the mild/moderate group. **(B)** The type 2 cytokine IL-4 appeared as the most characteristic principal mediator of the mild/moderate group, in addition to being identified as a key inflammatory mediators identified in the DyNA (see Fig 6A).

We next sought to characterize subsets of mediators in the form of orthogonal normalized linear combinations of the original inflammatory mediator variables, called principal components. This approach allows us to identify principal components of severe vs. mild/moderate extremity injury groups, thereby allowing us to infer principal characteristics of each inflammatory response [50, 52] (Fig 7). Overall, the PCA of the severe injury group exhibited more components than that of the mild/moderate injury group (9 vs. 7, respectively), as well as having a slightly greater overall magnitude (~0.23 vs. 0.20, respectively). The PCA from day 0–7 identified IL-1β and its antagonist IL-1RA as leading principal mediators in the severe extremity injury group but not in the mild/moderate group. Inversely, IL-4, IL-7, IL-13, and sIL-2Rα were leading components in the mild-moderate sub-cohort. Interestingly, IL-4 and IL-13, well-established type 2 cytokines associated with tissue healing as well as being two of the key inflammatory mediators identified in the DyNA, appeared as principal mediators in the mild/moderate group. IL-4 and IL-13, along with sIL-2Rα, were also identified as part of a dynamic inflammation structure by day 7 in the mild/moderate injury group. Both sub-cohorts shared IL-22 and IL-23 as principal mediators, in concordance with the DyNA results.

## Discussion

Severe extremity trauma induces an inflammatory response that contributes to both early and delayed complications, muscle necrosis, and ischemia reperfusion injury [12]. We and others have used both data-driven and mechanistic computational modeling approaches to address this complexity and to gain both basic and translational insights into trauma, hemorrhage, and related phenomena such as sepsis [12, 56]. Our goal in the present study was to examine the association between the clinical outcomes and the early, dynamic, systemic acute inflammatory response in the setting of major bone/soft tissue injury in a manner that would allow for the least degree of ambiguity while still reflecting the reality and diversity of clinical outcomes. In addition to confirming multiple prior observations about the clinical impact of different degrees of extremity injury severity in blunt trauma patients, we correlated these outcomes with differential inflammatory trajectories and dynamic networks.

The most striking finding of this study was the disparate trajectories of inflammation network complexity between the two groups. The gradually increasing network connectivity in the severely injured sub-cohort would suggest that those patients experiencing significant bone/soft tissue damage mount an inflammatory response to the damage which does not resolve even up to 7 days following the insult. In fact, the nature of the injuries is such that the level of inflammation rapidly trends upward over 7 days. This may be due to impact of the nature of bone and soft tissue (muscle, skin and subcutaneous tissues) damage and repair processes that lead to a prolonged release of inflammatory mediators. The progression of interconnected innate and lymphoid mediators over 7 days suggests that the inflammation resulting from the damaged extremities is self-sustaining, similar to the dynamic network connectivity pattern we have reported recently for blunt trauma non-survivors [46].

This network phenotype was associated with significantly elevated levels of several chemokines and cytokines. These included the chemokines IL-8, MIG, IP-10, and MCP-1; we have reported elevations of these mediators in a separate study of extremity fracture patients [57]. We have also demonstrated previously that two of these chemokines (MCP-1 and IP-10) are biomarkers of adverse outcomes in trauma [48, 51]. Circulating levels of the cytokine IL-6 were statistically significantly different between groups by AUC analysis, again in line with a previous study in a separate cohort of extremity fracture patients [57]. The role of IL-6 in this setting may be quite central, since recent studies have reported improved fracture healing in an animal model of bone fracture following administration of neutralizing antibodies directed against the IL-6 receptor [58]. Notably, IL-6 was not connected to the network of other inflammatory mediators in our analysis, despite being elevated significantly in the severe extremity injury patients; this feature (“elevated but not connected”) was noted in our initial DyNA study comparing mouse trauma/hemorrhage vs. trauma alone [50]. Other mediators (IL-7, eotaxin, and MIP-1α), in contrast, were present at lower levels in the systemic circulation of patients with severe extremity injuries as compared to those with mild/moderate injury. It is tempting to speculate that this difference in circulating IL-7 is, in part, related to the higher rate of nosocomial infections in the severe extremity fracture group as compared to the mild/moderate injury group, given that circulating IL-7 levels are reduced in sepsis patients [59] and that administration of IL-7 can restore lymphocyte functions in the setting of sepsis [60].

The apparent self-sustaining, pro-inflammatory phenotype of the severe extremity injury group is in distinct contrast to the type of inflammation discerned in the mild/moderate injury severity group. Although the early inflammatory networks in this group are defined by more highly connected innate and lymphoid mediators, the stepwise regression of these interconnections over 7 days was correlated with the overall improved clinical outcomes of the mild/moderate cohort. Principal Component Analysis suggested a central role for type 2 inflammatory mediators such as IL-4 and IL-13, supporting the concept of an inflammatory milieu biased towards resolution of inflammation. Furthermore, the inflammatory mediators within the DyNA networks suggested a characteristic, lymphoid-predominant, core inflammatory network of mediators which was remarkably comparable to the network associated previously with survival in blunt trauma patients up to 7 days post-injury [46].

Taken together, these findings point to a potential tipping point at approximately days 2–4 post-injury, at which the early, highly-connected networks of innate and lymphoid mediators in both groups appear to be set on a trajectory of either self-sustaining pathologic inflammation associated with severe extremity injury vs. self-resolving reparative inflammation in the absence of extremity injury. Given the highly matched nature of these sub-cohorts, the attention given to confounding variables, and the similarly sustained levels of injury reflected by matched ISS, we hypothesize that a host of predetermined genetic, epigenetic, and environmental factors exist within the population that predispose patients for a given inflammatory trajectory.

Open fractures and surgical fixation of more severe extremity injuries likely account for the greater degrees of blood loss over time, as surgical debridements and complex fracture reductions are often more extensive. However, admission hemoglobin and coagulation parameters were essentially identical between the groups, indicating that initial blood loss was likely similar between the groups. Interestingly, while initial bleeding was similar, patients with severe extremity injuries had significantly greater levels of anaerobic metabolism at the time of injury. This may have resulted from limbs with greater volume of devitalized tissue or from limbs with greater levels of ischemia in the severe group, but this is not known. The data do indicate that for equivalent overall injury severity indices, patients with severe extremity trauma have increased anaerobic metabolism and poor early immunologic orchestration. Differences in the immunologic response may have resulted from increases in anaerobic metabolism or may be associated with limb trauma severity.

There are several potential clinical implications of our work. Despite use of early, definitive fracture interventions in physiologically stable trauma patients, and application of “damage control” temporizing (external fixation) measures in unstable (shock, acidosis, hypothermia, severe head or chest injury) trauma patients, the risk of complications such as wound infection, organ dysfunction, and prolonged hospital stays remain high in polytrauma patients with orthopaedic injuries [61, 62]. There is mounting evidence that post-injury immunologic dysregulation may account for disparate clinical courses [12–14]. Improved comprehension of temporal post-traumatic inflammatory profiles in extremity-injured patients may guide orthopedic surgeons and trauma surgeons when deciding on the timing and magnitude of surgical fracture fixation. Although data from this investigation are not capable of directly guiding orthopedic management, they suggest that early orchestration of the immunologic response may distinguish the severity of the extremity injury and the overall effect of the extremity injury on the patient. In addition, differences in immunologic orchestration that were observed in this study occurred very early in the clinical course. Taken together, early profiling of dynamic immunologic networks has the potential to inform management decisions pertaining to extremity injuries. It is possible that early fracture stabilization may mitigate early dysregulated inflammation observed in this study in the form of self-sustaining inflammatory networks in patients with severe injuries. In contrast, early identification of at-risk patients via immune profiling may influence surgeons to delay definitive fracture procedures until a dysregulated immunologic response has resolved. Immunologic response information, which can now be measured in a relevant time frame, offers another set of data to inform extremity fracture surgery and other titrated care decisions. Further investigations should focus on specific injury patterns and the impact of surgical timing on immunologic networks.

As in our previous work [7], we recognize that there are several limitations in our study. First, this study was performed at a single, Level I trauma center and thus may not be generalizable or pertinent to other centers with differing admission demographics, injury characteristics, or management practices. This issue warrants additional, similar studies in other trauma centers to validate the results suggested from the current study. Another important limitation of this retrospective study is the potential impact of blood transfusion and surgical interventions on the temporal dynamics of the inflammatory response. We note that these interventions are by necessity an intrinsic element of clinical care for management of trauma patients with evidence of blood loss. This differential requirement for transfusion may impact the systemic inflammatory response. We were also limited in gathering information regarding the detailed surgical interventions in term of fracture reduction methods and procedure. Moreover, the number of inflammatory mediators analyzed, which was limited to the number of mediators we could measure using commercially available Luminex bead sets. In this regard, while the inflammatory mediators that we have assayed interrogate a broad array of innate and adaptive immune pathways known to be modulated in our broader trauma patient population, it is formally possible, though in our opinion unlikely, that a completely different response is triggered as a function of severe extremity injury as compared to mild/moderate extremity injury. Future studies should include more in-depth immune profiling to include additional components of the immune response. Another limitation concerns the potentially confounding effect of age in interpreting the results, given recent findings about the differential systemic inflammatory responses in older vs. younger trauma patients [37–41]. Finally, we note that data-driven modeling relies on available data, and as such depends on the quality of those data. These tools do not provide any direct mechanistic information about the biology beneath it, however, they are suggesting possible interactions among inflammatory mediators.

In conclusion, the current study demonstrates the presence of differential, extremity injury-graded early systemic inflammatory responses. These inflammatory responses are associated tightly with significantly differential clinical outcomes. Our results suggest that severe extremity/soft tissue injury can drive a differential inflammation program associated with self-sustaining inflammation and worse clinical outcomes, as compared to mild/moderate soft tissue injury, which is associated with a core network of lymphoid inflammatory mediators and self-resolving inflammation. We suggest that an approach combining stringently-matched cohorts, extensive sampling, and computational modeling can be used to gain similar insights into other aspects of acute illness.

## Supporting information

S1 Fig Original DyNA outputs. Trauma patients were recruited following IRB approval and informed consent. Plasma was obtained at multiple time points and analyzed for the presence of 31 inflammatory mediators in highly-matched sub-cohorts of patients with severe vs. mild/moderate extremity injury, followed by Dynamic Network Analysis (DyNA) as described in the *Materials and Methods*.
(PDF)

S2 Fig Comparison of individual components of the Marshall MODScore in mild/moderate vs. severe extremity injury sub-cohorts (*P<0.05, analyzed by Two-Way ANOVA). (PDF)

S1 Table Inflammation biomarkers in the mild/moderate, and severe extremity injury sub-cohorts from time of injury up to 7 days. Trauma patients were recruited following IRB approval and informed consent. Plasma was obtained at multiple time points and analyzed for the presence of 27 inflammatory mediators in highly-matched sub-cohorts of patients with severe vs. mild/moderate extremity injury as described in the *Materials and Methods*.
(XLSX)

S2 Table A quantitative analysis of DyNA connectivity shows a 38.3% increase in the number of mediators connected in the severe injury group vs. the mild/moderate group. (DOCX)

## Abbreviations

MODS: Multiple Organ Dysfunction Syndrome
ICU: Intensive Care Unit
ISS: Injury Severity Score
AIS: Abbreviated Injury Scale
SI: Shock index
IL: Interleukin
IFN: Interferon
MCP-1: Monocyte Chemotactic Protein-1 (CCL2)
MIG: Monokine Inducible by Interferon-γ (CXCL9)
MIP-1α: Macrophage Inflammatory Protein-1α (CCL3)
TNF-α: Tumor Necrosis Factor-α
DyNA: Dynamic Network Analysis
