# Supplementary material for: Computational evidence for an early, amplified systemic inflammation program in polytrauma patients with severe extremity injuries
Source: PLoS One. 2019 Jun 4;14(6):e0217577. doi: 10.1371/journal.pone.0217577 (PMC6548366; doi:10.1371/journal.pone.0217577)

S1 Fig.

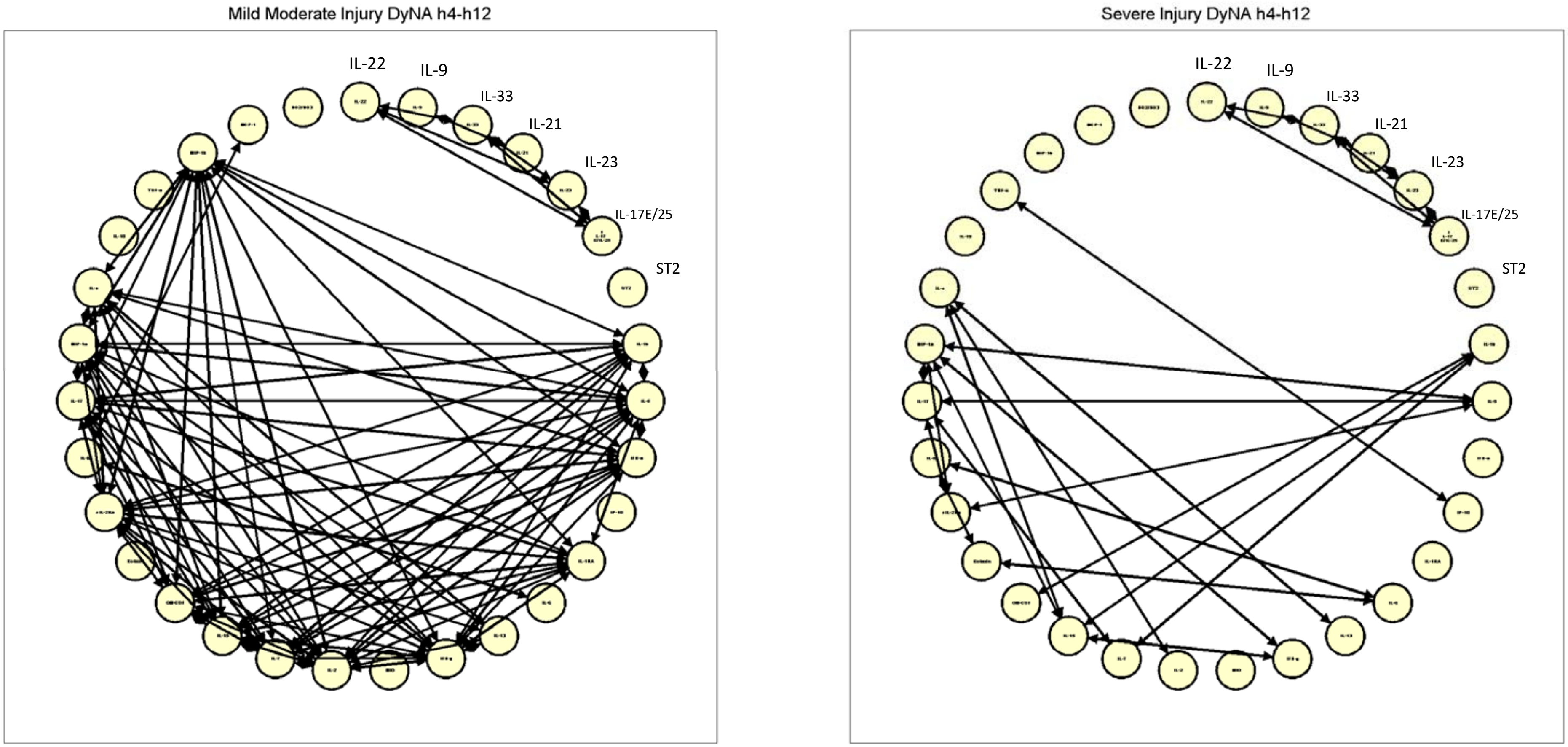
Mild Moderate Injury DyNA h4-h12
IL-22
IL-9
IL-33
IL-21
IL-23
IL-17E/25
ST2
Severe Injury DyNA h4-h12
IL-22
IL-9
IL-33
IL-21
IL-23
IL-17E/25
ST2

Mild Moderate Injury DyNA h8-h16

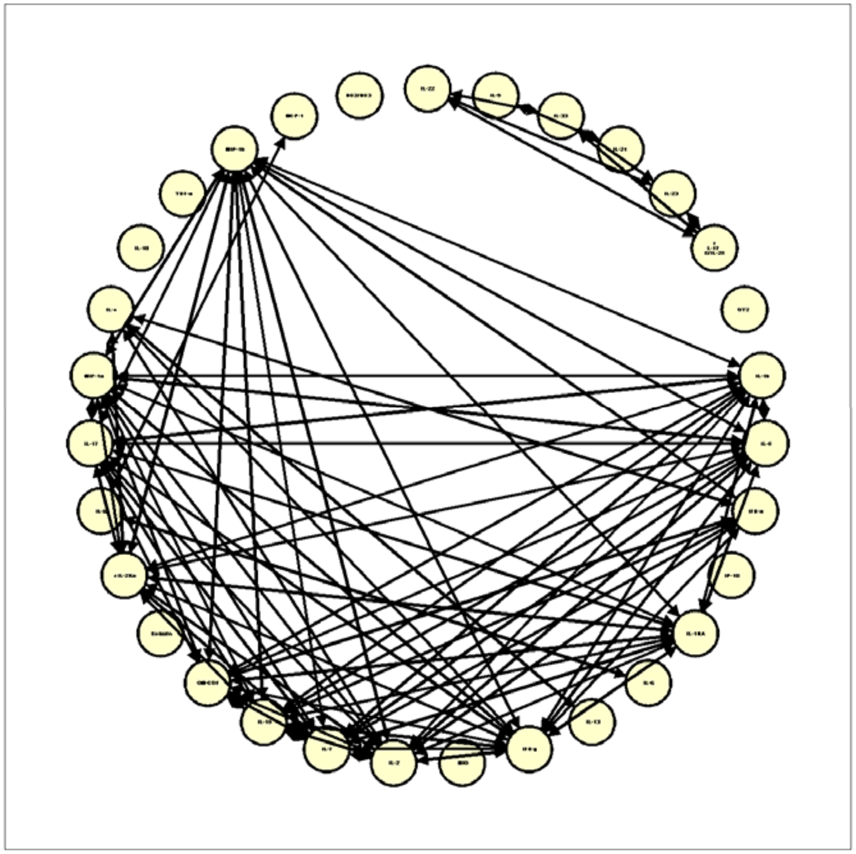

Severe Injury DyNA h8-h16

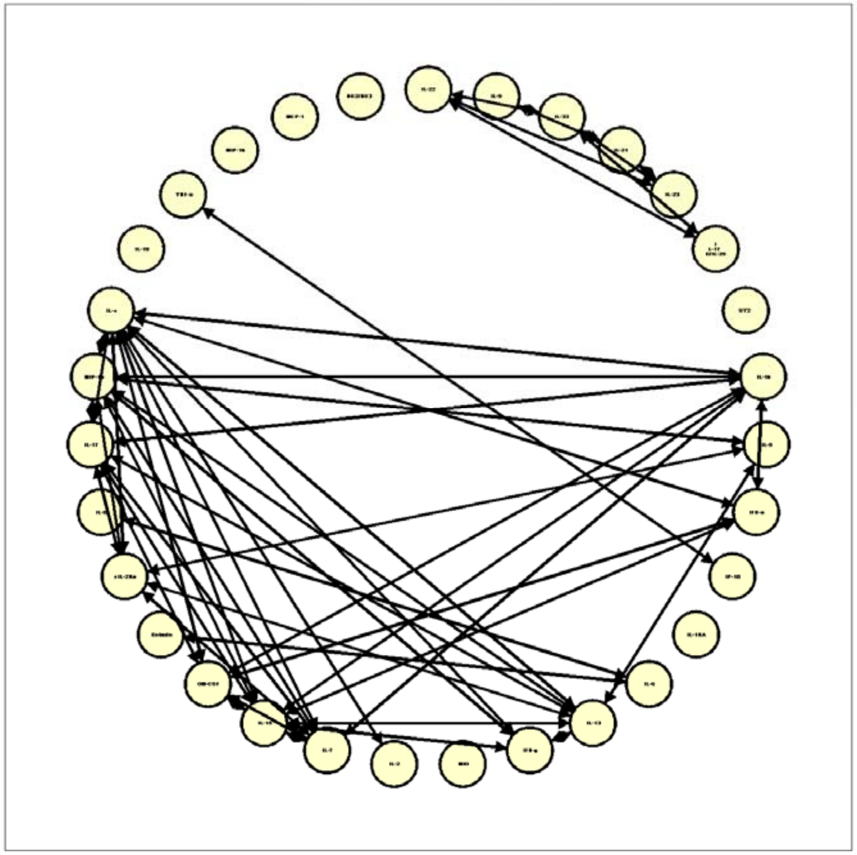

Mild Moderate Injury DyNA h12-h20

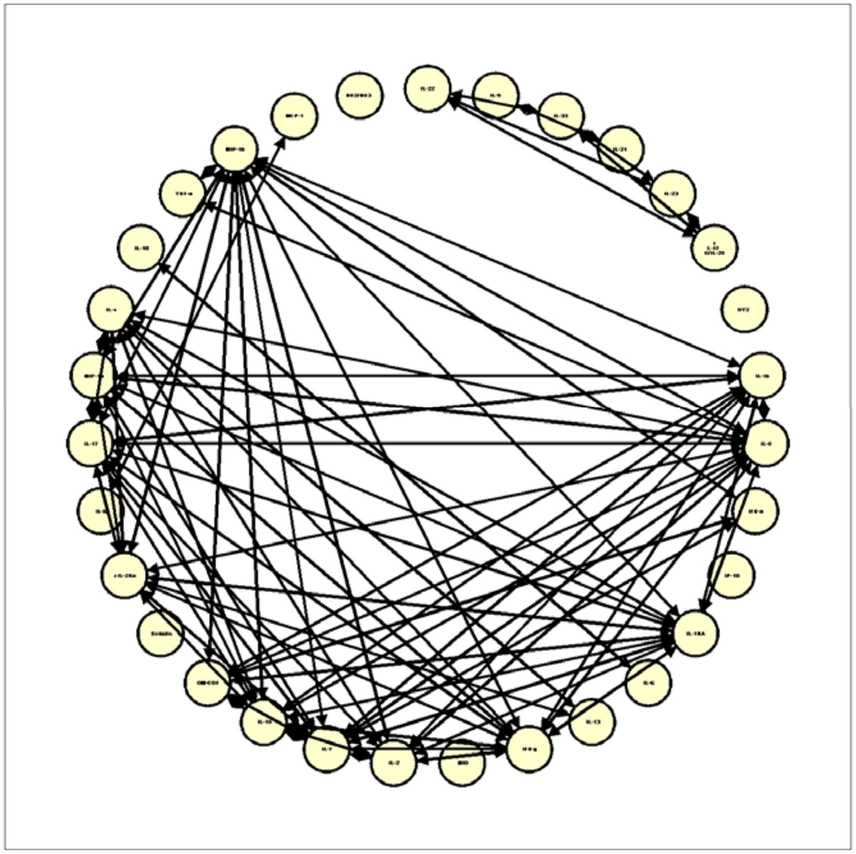

Severe Injury DyNA h12-h20

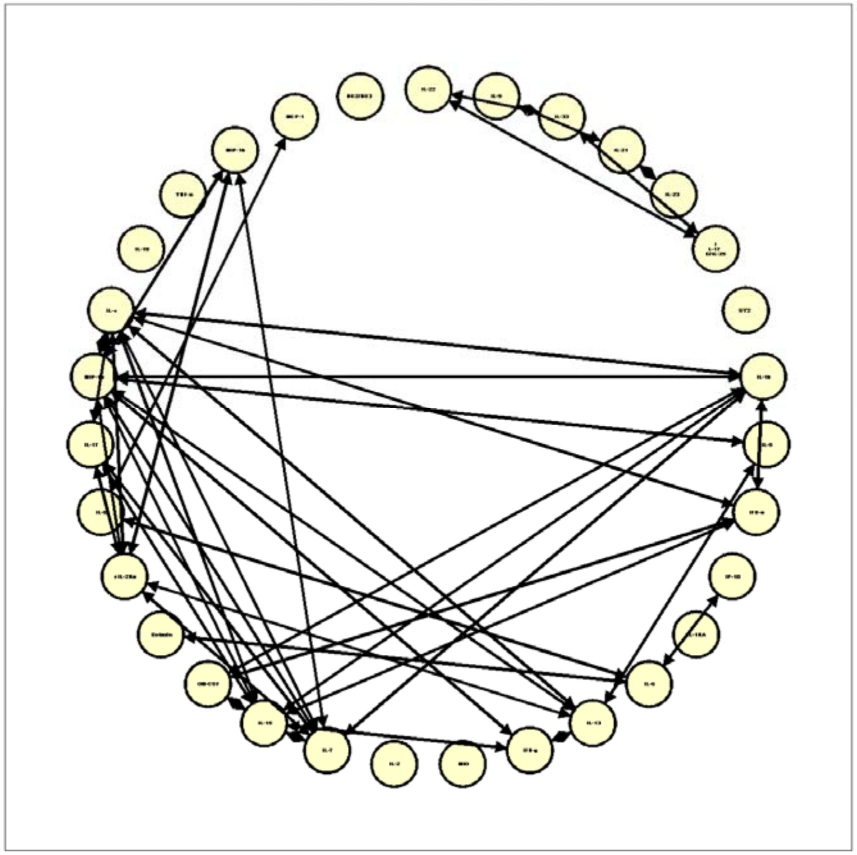

Mild Moderate Injury DyNA h16-h24

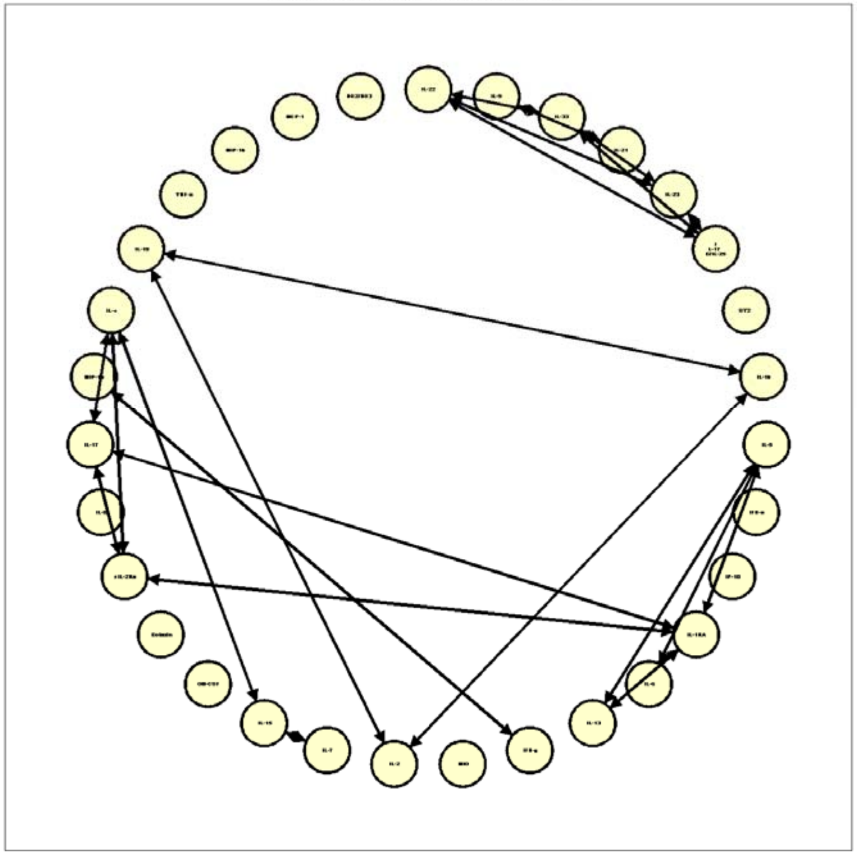

Severe Injury DyNA h16-h24

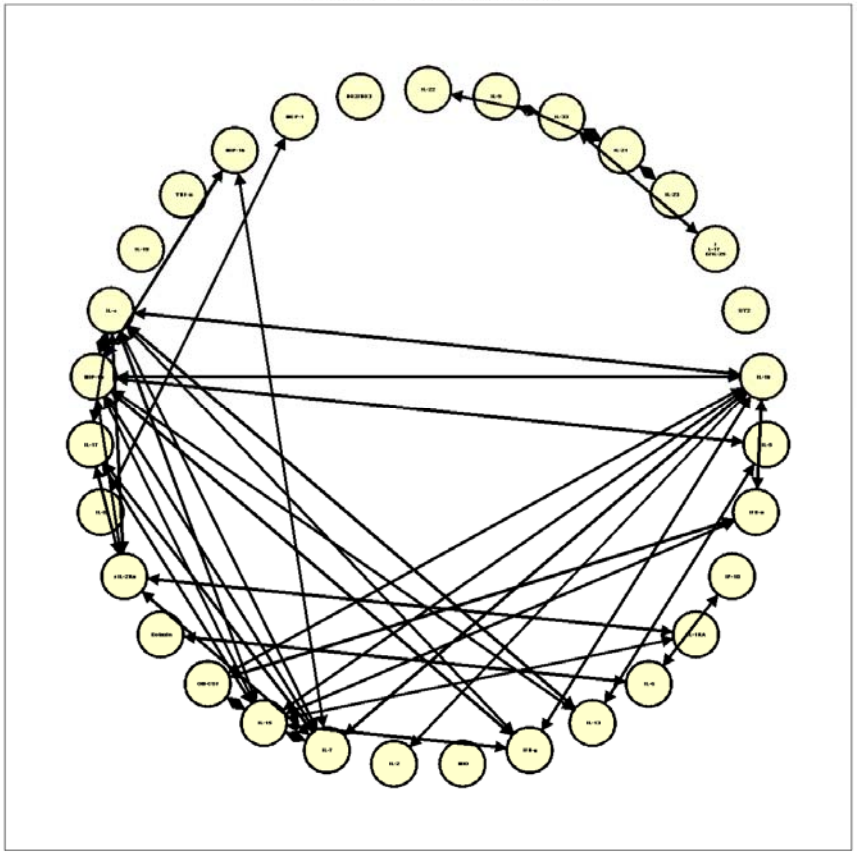

Mild Moderate Injury DyNA h20-D1

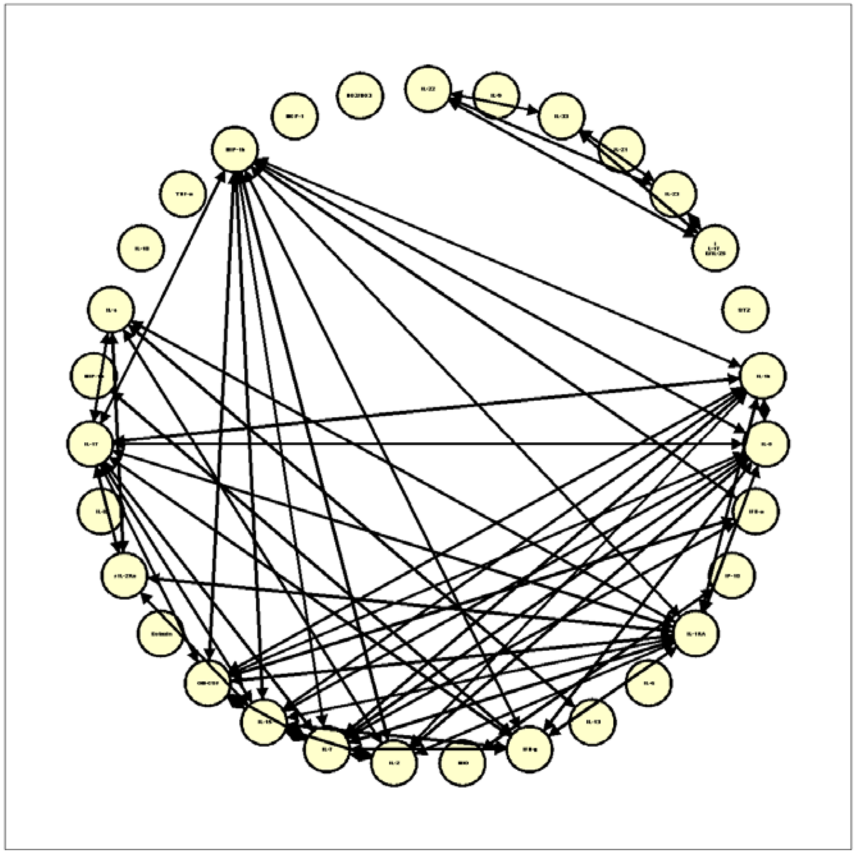

Severe Injury DyNA h20-D1

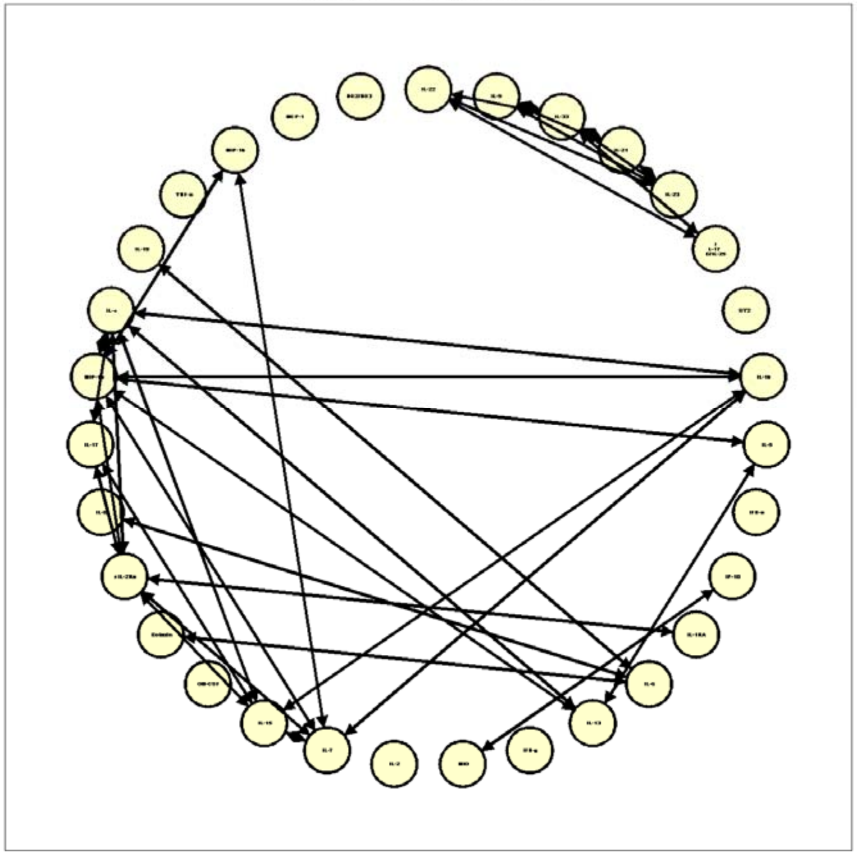

Mild Moderate Injury DyNA h24-D2

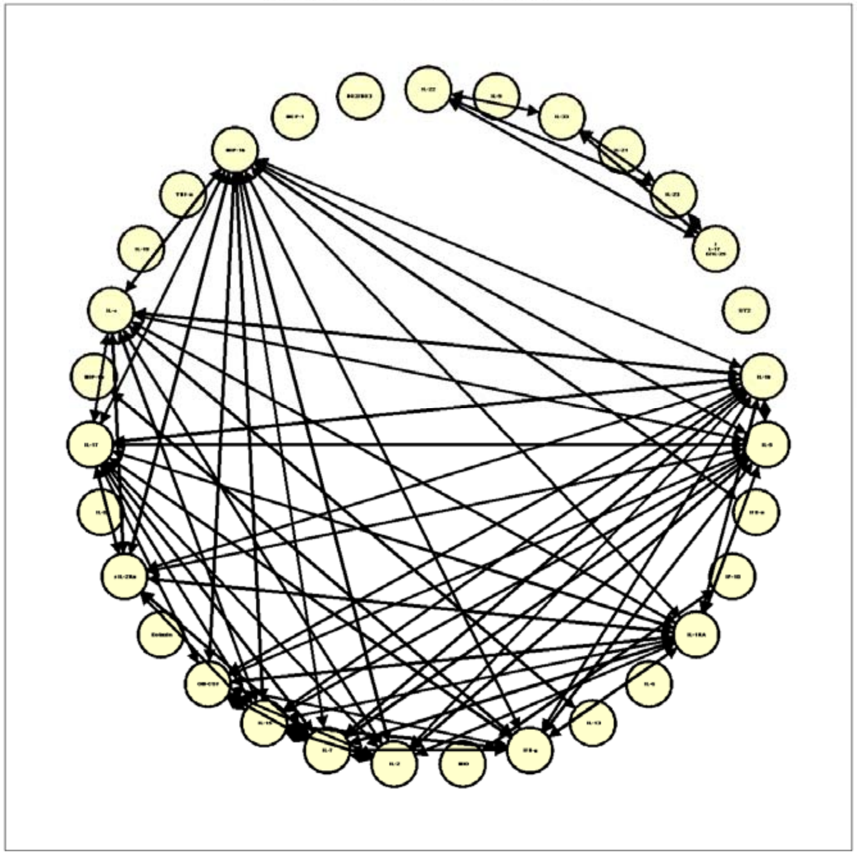

Severe Injury DyNA h24-D2

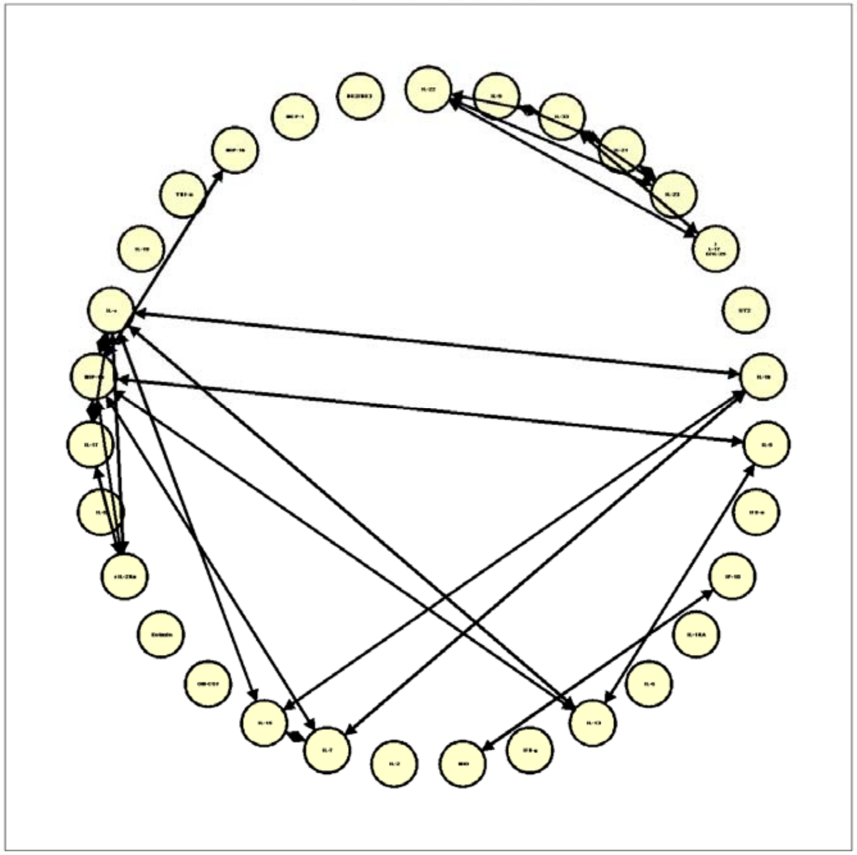

Mild Moderate Injury DyNA D1-D3

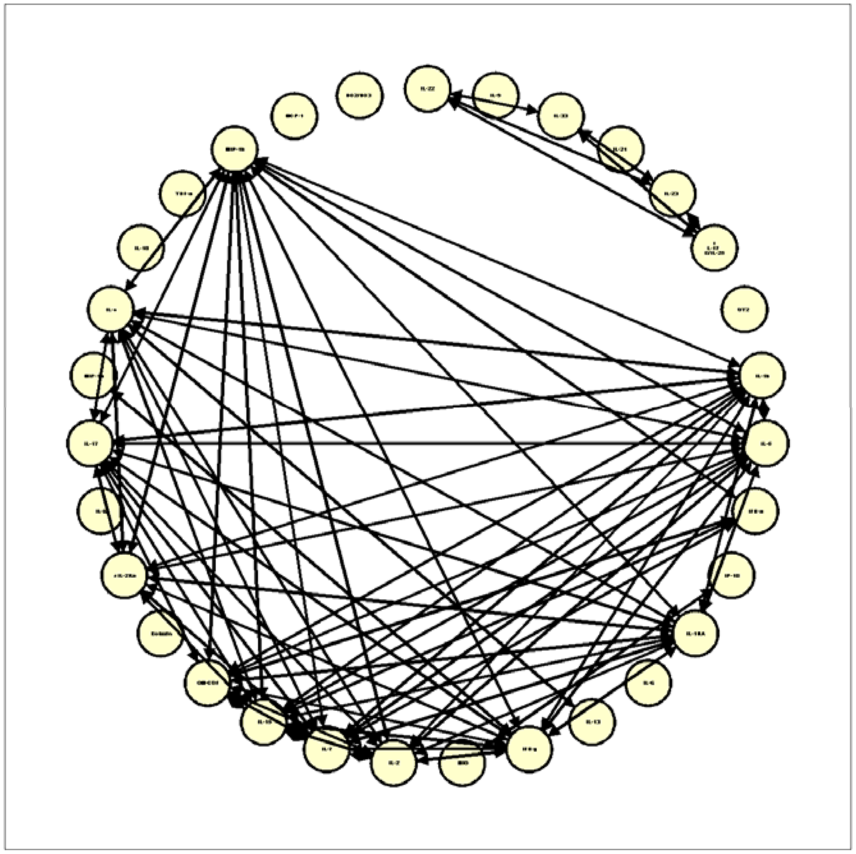

Severe Injury DyNA D1-D3

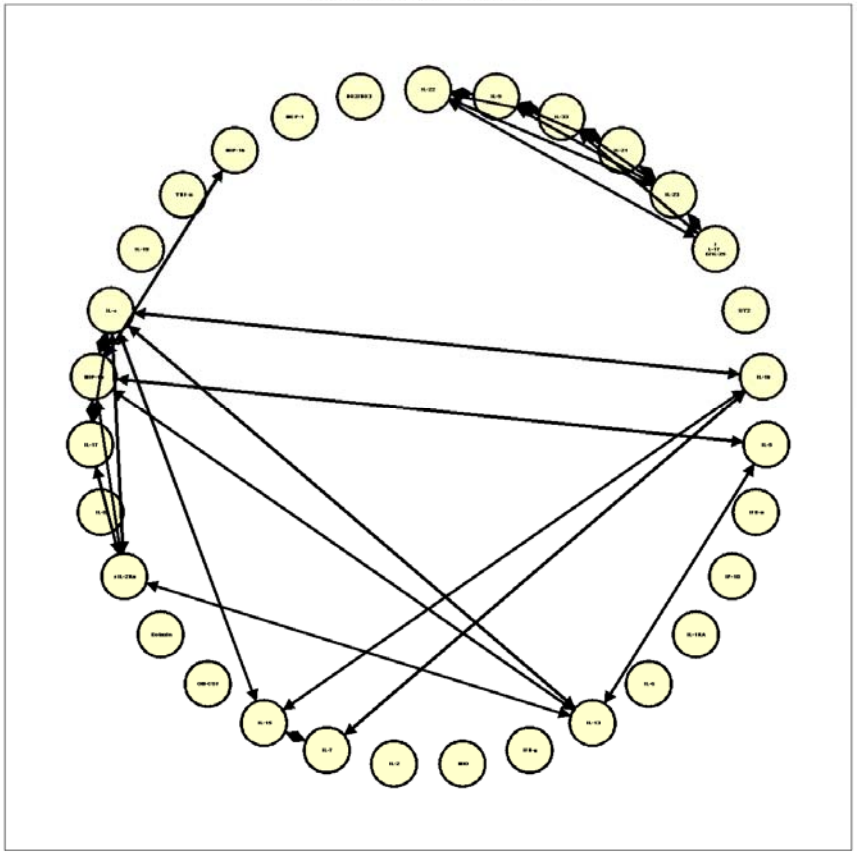

Mild Moderate Injury DyNA D2-D4

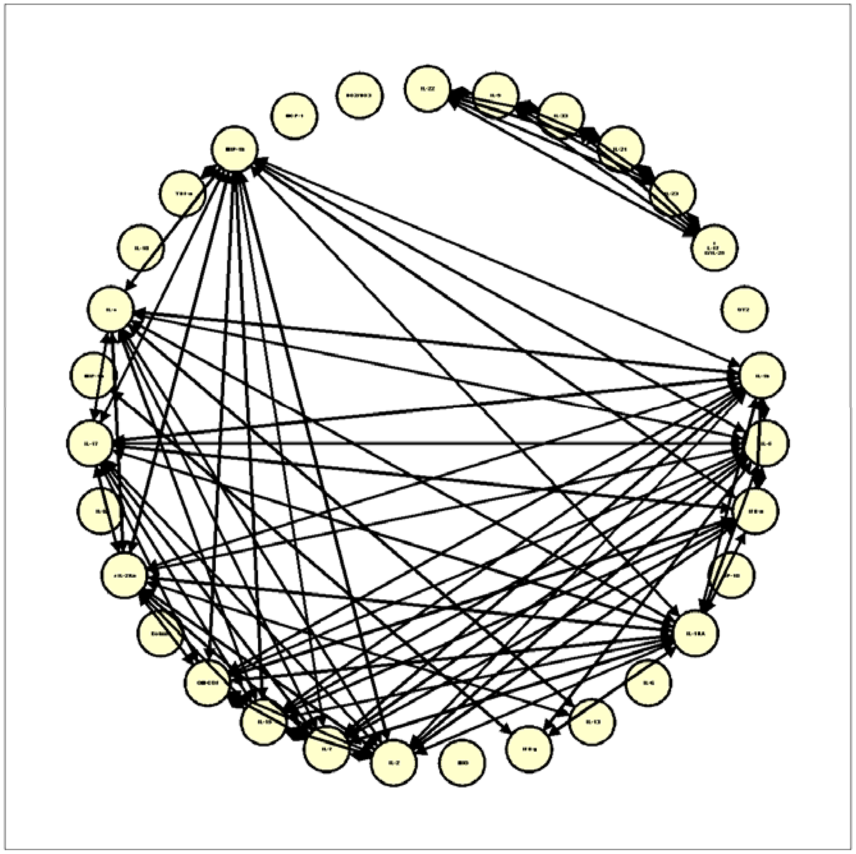

Severe Injury DyNA D2-D4

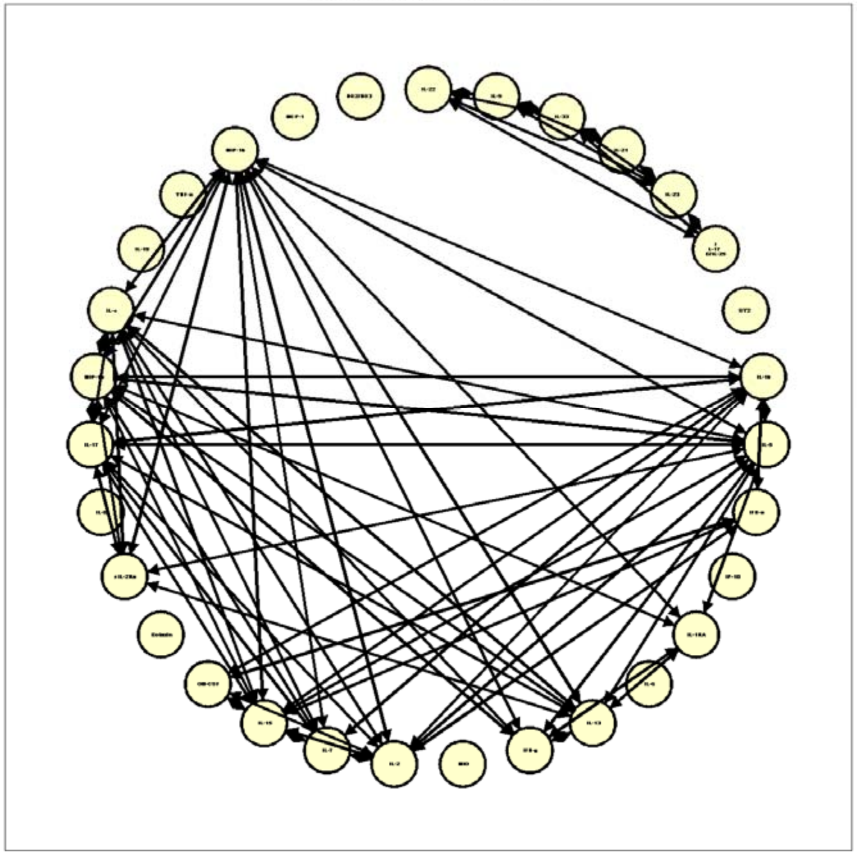

Mild Moderate Injury DyNA D3-D5

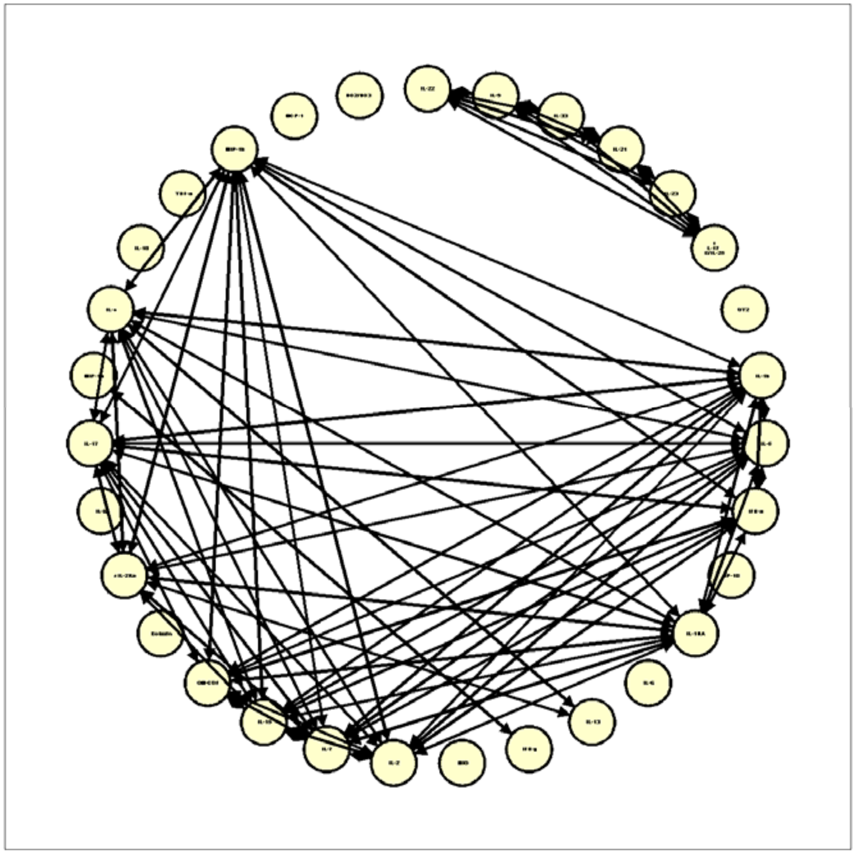

Severe Injury DyNA D3-D5

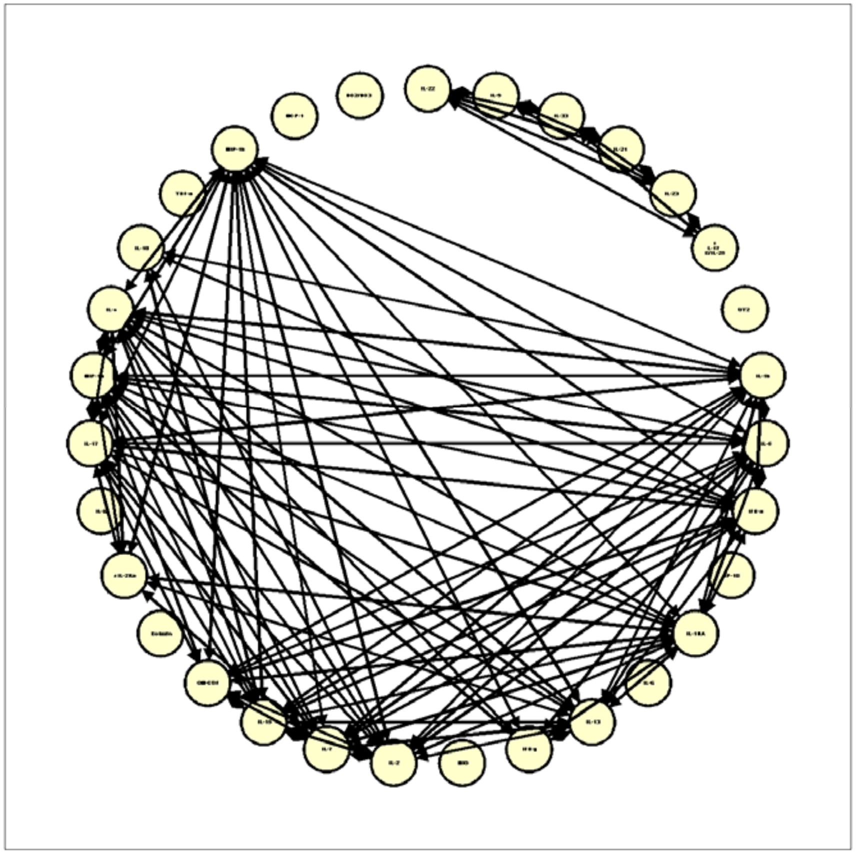

Mild Moderate Injury DyNA D4-D6

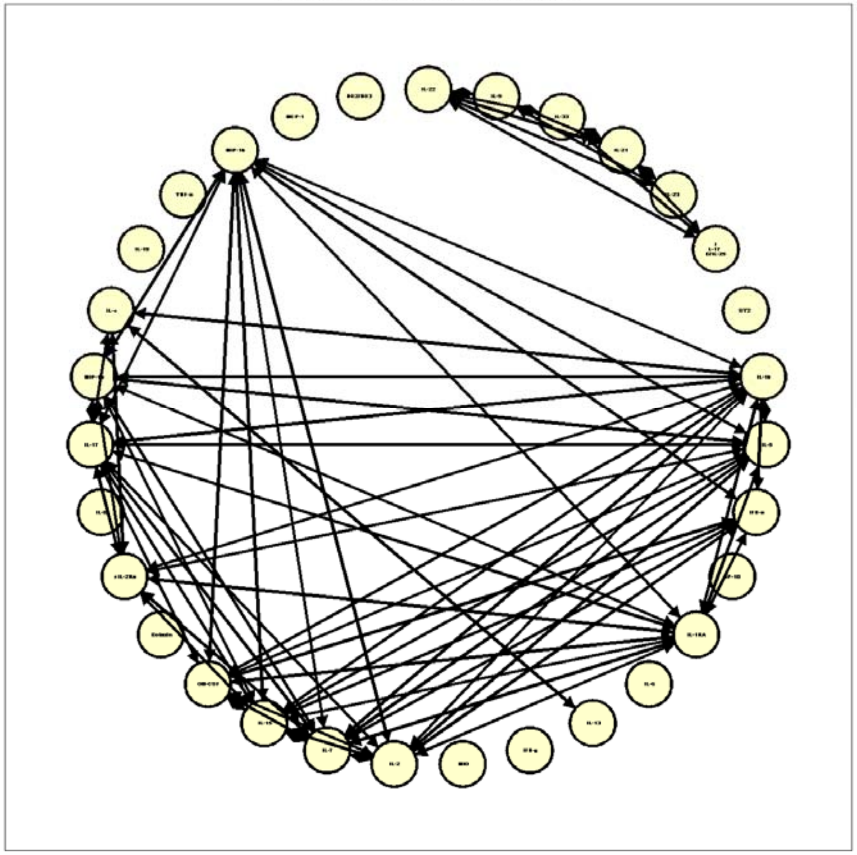

Severe Injury DyNA D4-D6

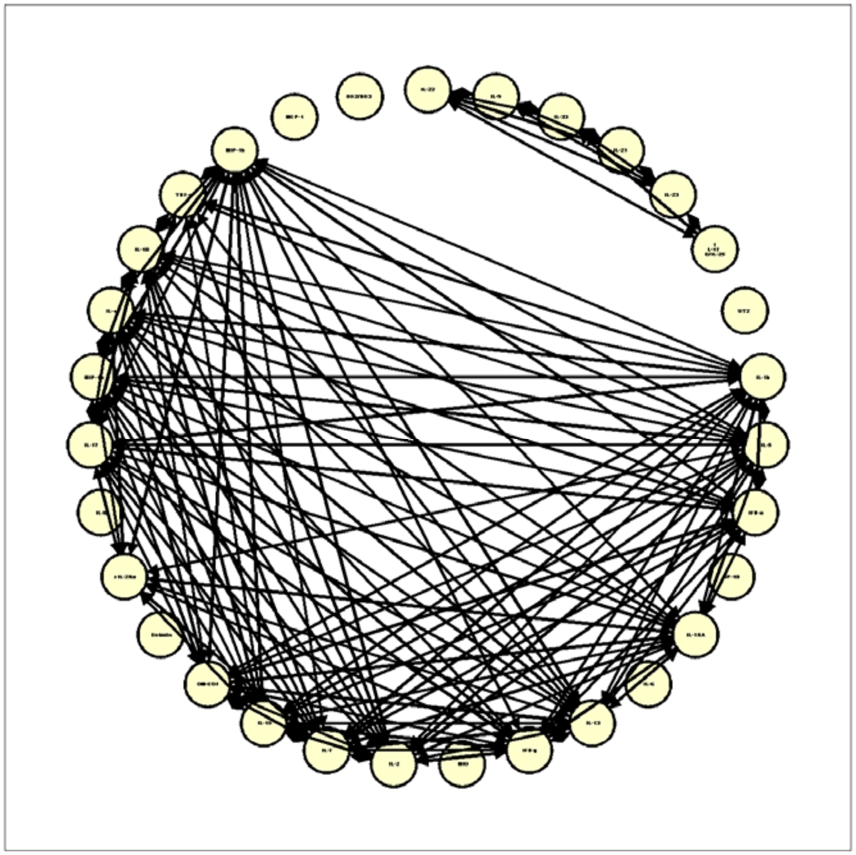

Mild Moderate Injury DyNA D5-D7

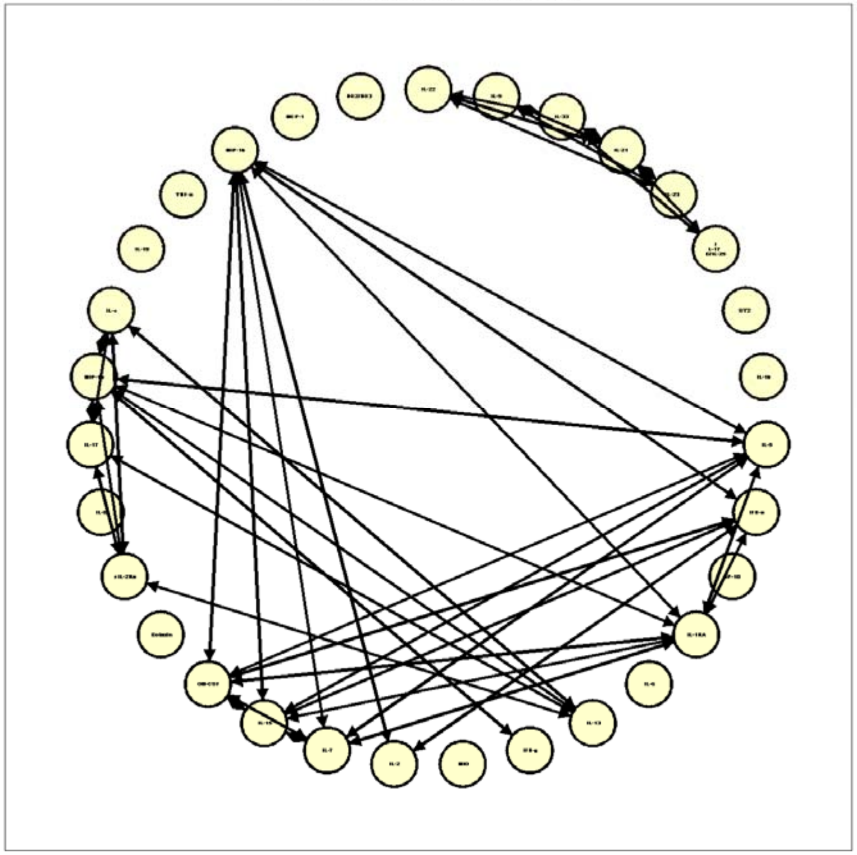

Severe Injury DyNA D5-D7

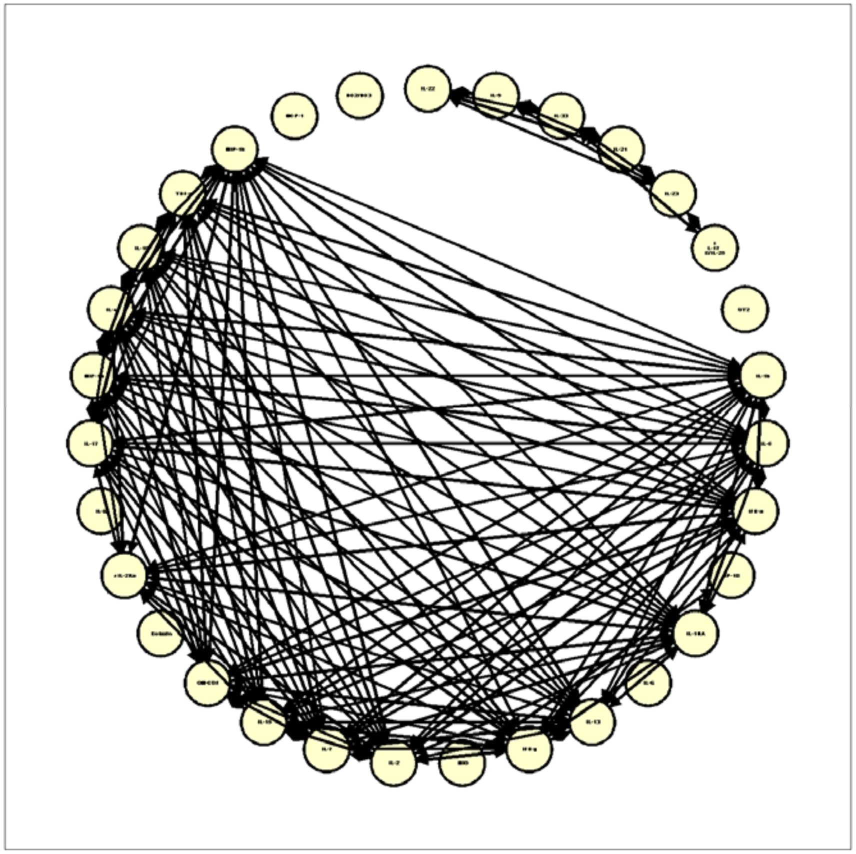

Supplement: S1 Fig — Trauma patients were recruited following IRB approval and informed consent. Plasma was obtained at multiple time points and analyzed for the presence of 31 inflammatory mediators in highly-matched sub-cohorts of patients with severe vs. mild/moderate extremity injury, followed by Dynamic Network Analysis (DyNA) as described in the Materials and Methods. (PDF) [file pone.0217577.s001.pdf]
