# Supplementary figures and images for: Computational evidence for an early, amplified systemic inflammation program in polytrauma patients with severe extremity injuries
Source: PLoS One. 2019 Jun 4;14(6):e0217577. doi: 10.1371/journal.pone.0217577 (PMC6548366; doi:10.1371/journal.pone.0217577)

S2 Fig.

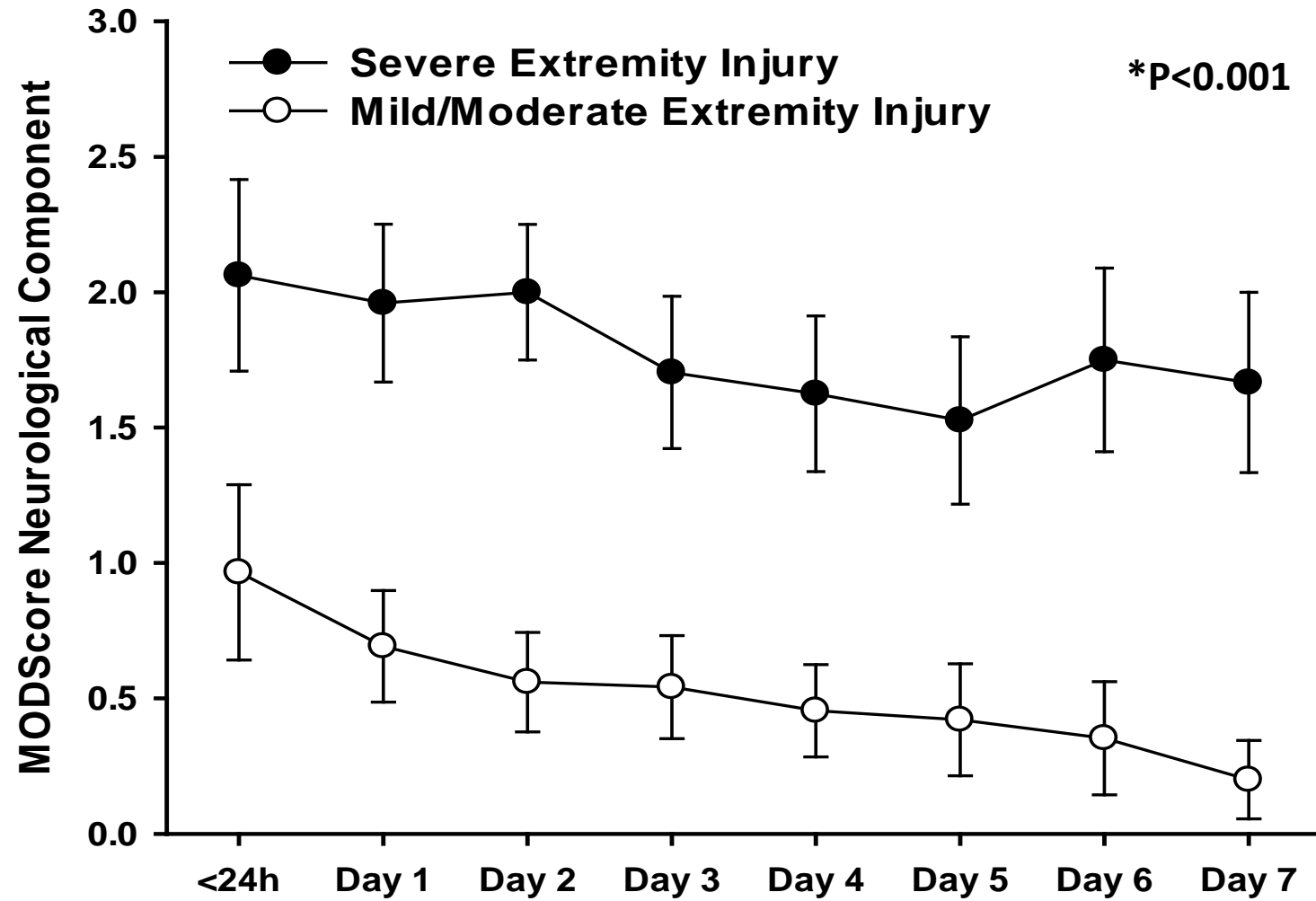

S2 Fig.

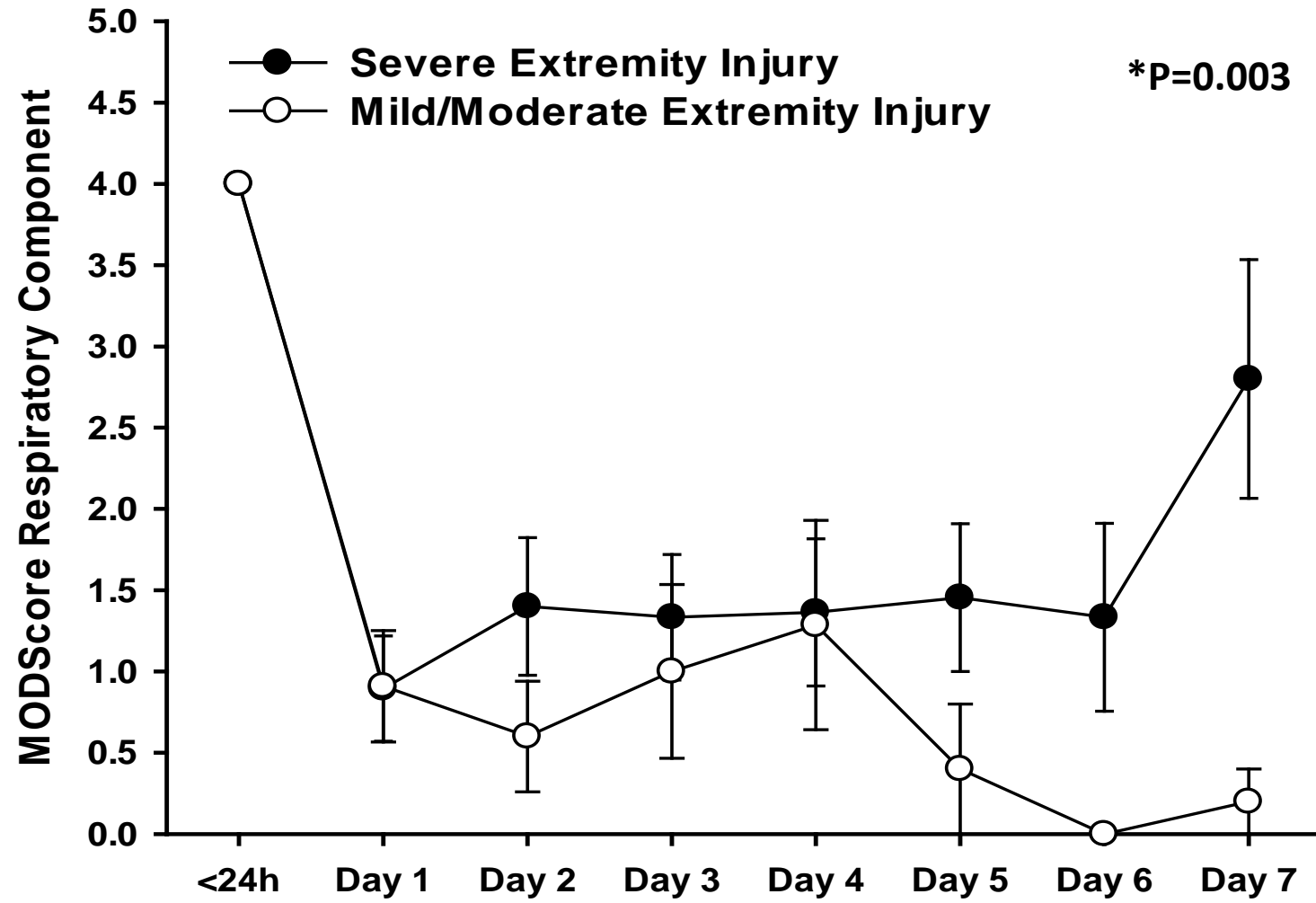

S2 Fig.

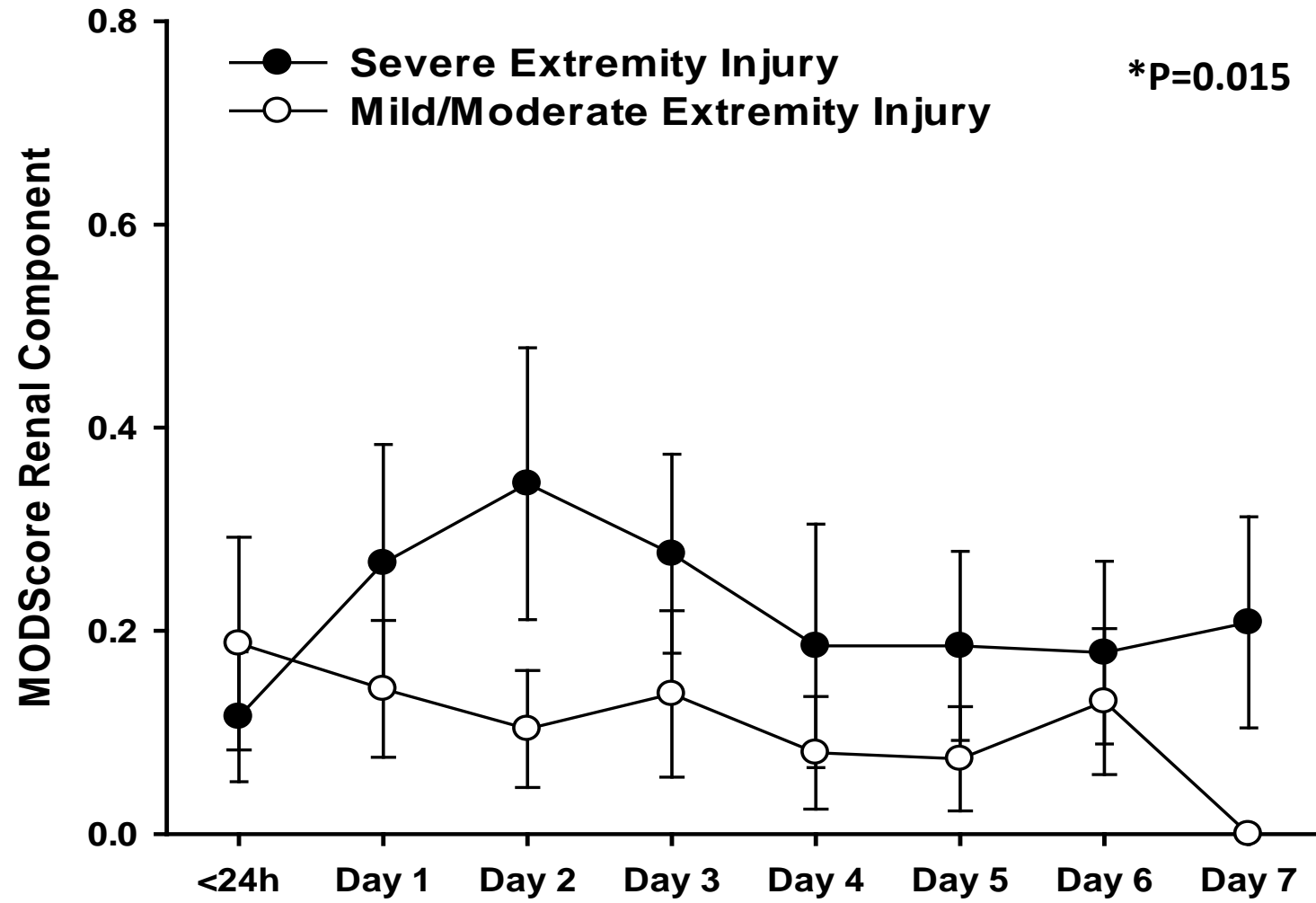

S2 Fig.

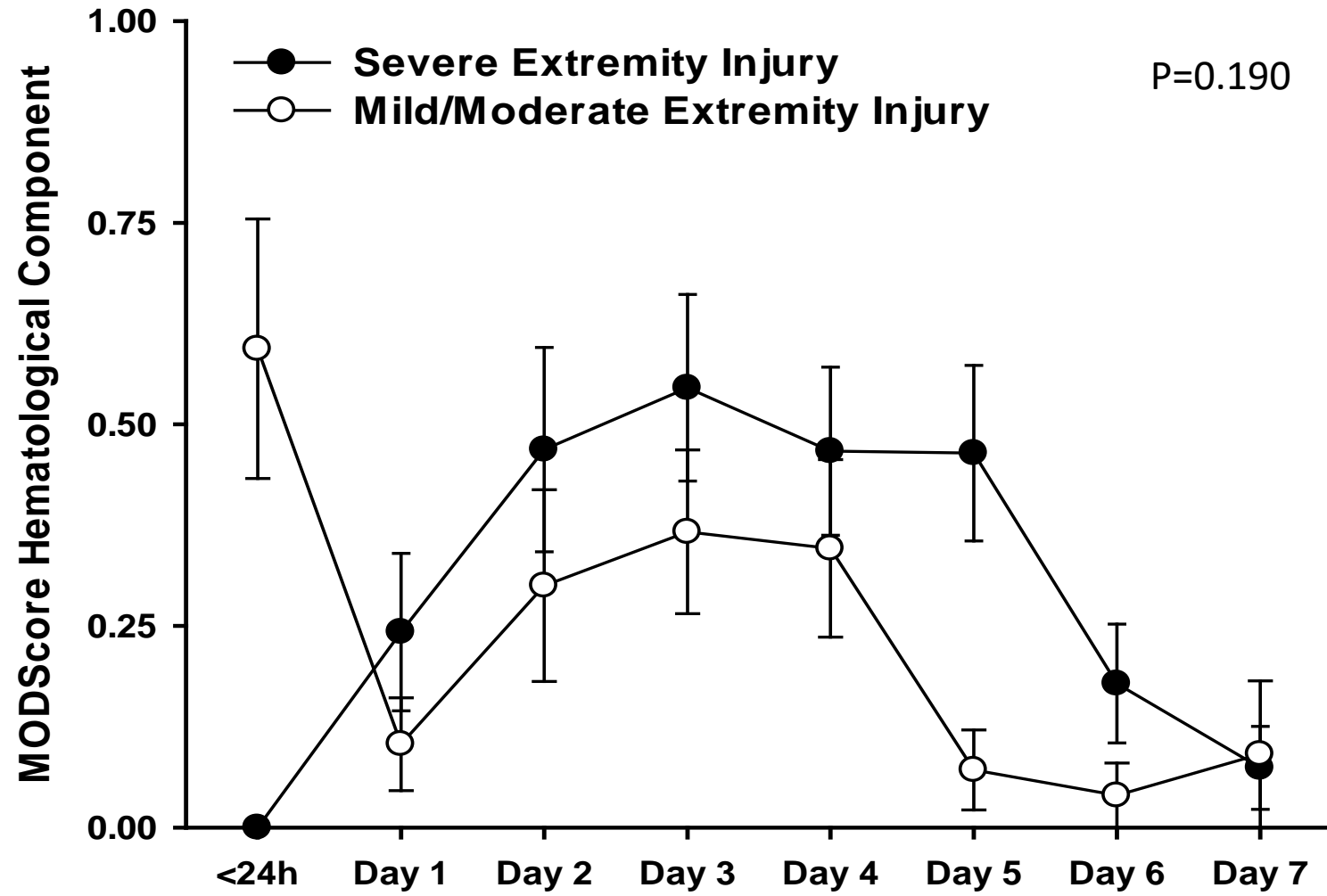

S2 Fig.

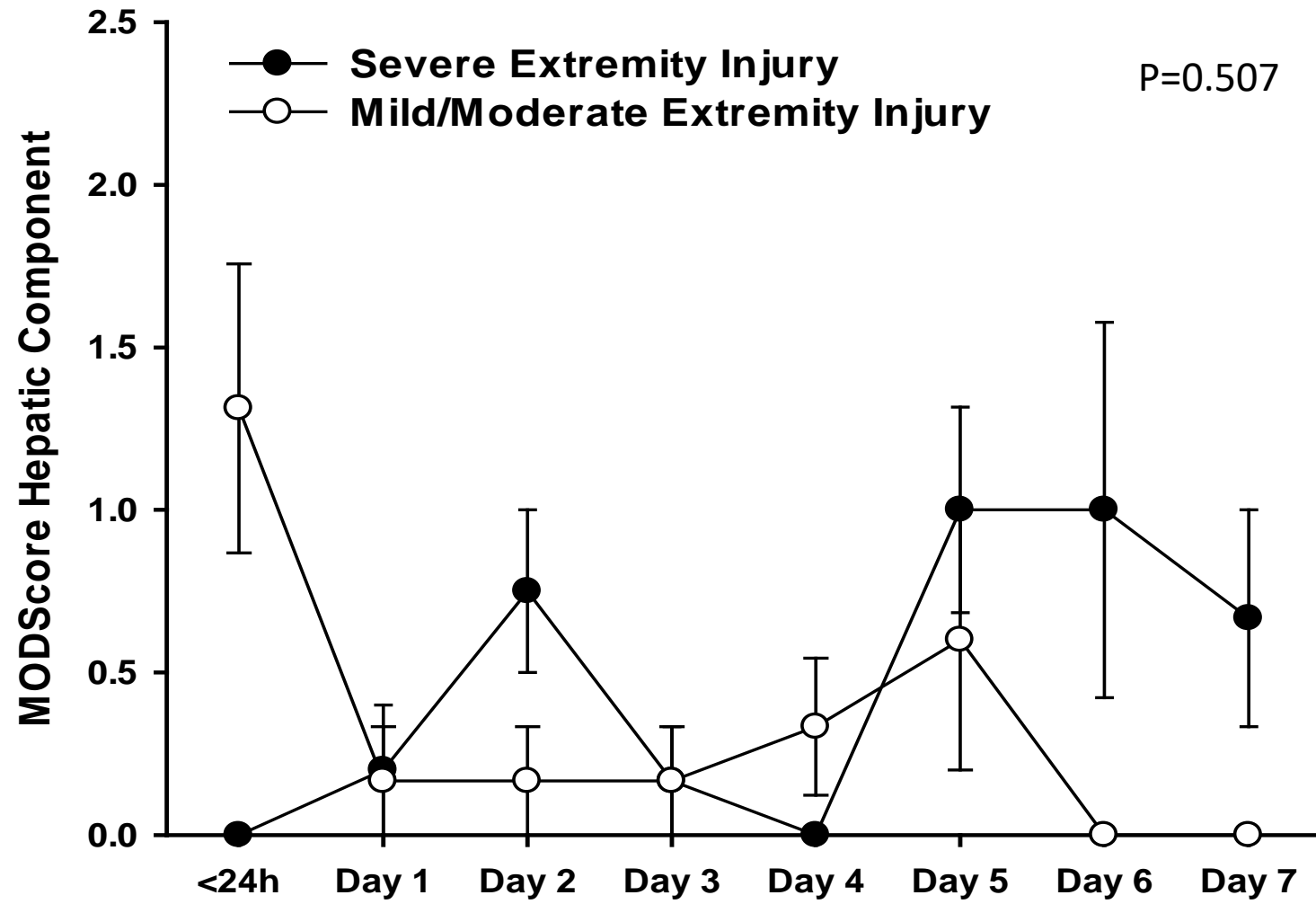

S2 Fig.

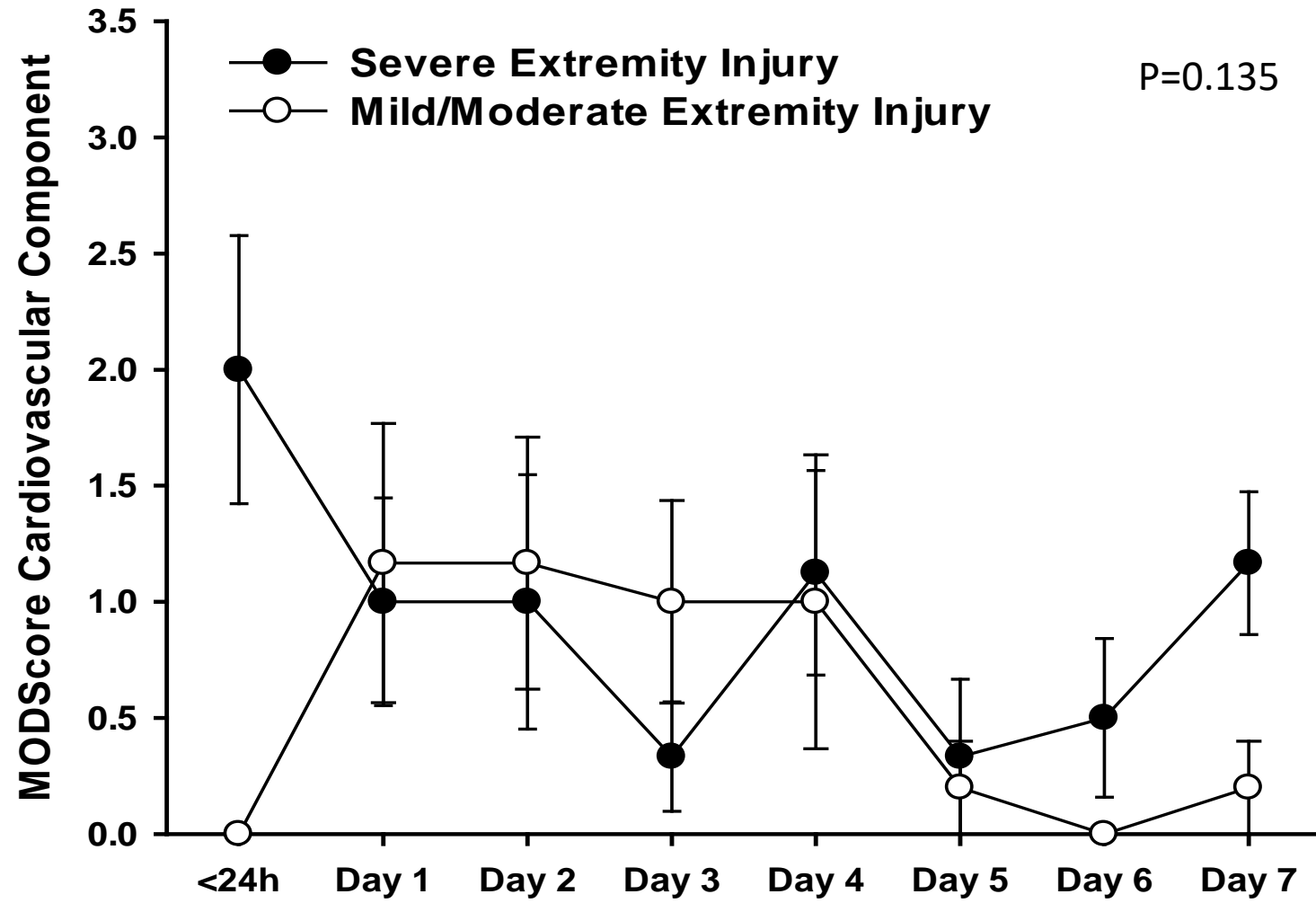

Supplement: S2 Fig — (PDF) [file pone.0217577.s002.pdf]
