# Supplementary material for: Computational evidence for an early, amplified systemic inflammation program in polytrauma patients with severe extremity injuries
Source: PLoS One. 2019 Jun 4;14(6):e0217577. doi: 10.1371/journal.pone.0217577 (PMC6548366; doi:10.1371/journal.pone.0217577)
Supplement: S2 Table — (DOCX) [file pone.0217577.s004.docx]

| **Number of Connections Day 1-7** | | | | |
| --- | --- | --- | --- | --- |
| **Mild-Moderate** | |  | **Severe** |  |
| MIP-1b | 50 |  | IL-4 | 58 |
| IL-1Ra | 49 |  | MIP-1b | 56 |
| IL-15 | 48 |  | IL-5 | 53 |
| IL-5 | 47 |  | IL-13 | 53 |
| IL-17 | 47 |  | IL-17 | 53 |
| IL-1b | 42 |  | IL-1b | 52 |
| IL-2 | 41 |  | IL-15 | 52 |
| GM-CSF | 41 |  | IL-2 | 49 |
| IL-2R | 38 |  | IL-7 | 49 |
| IL-4 | 38 |  | IL-1Ra | 47 |
| IFN-a | 29 |  | IFN-a | 45 |
| IL-7 | 26 |  | GM-CSF | 43 |
| IFN-g | 15 |  | IFN-g | 36 |
| IL-13 | 11 |  | IL-2R | 36 |
| IL-8 | 5 |  | IL-10 | 26 |
| MIP-1A | 3 |  | TNF-a | 22 |
| TNF-a | 2 |  | MIP-1A | 4 |
| IP-10 | 0 |  | IP-10 | 1 |
| IL-6 | 0 |  | MIG | 1 |
| MIG | 0 |  | IL-6 | 0 |
| Eotaxin | 0 |  | Eotaxin | 0 |
| IL-10 | 0 |  | IL-8 | 0 |
| MCP-1 | 0 |  | MCP-1 | 0 |
| NO2/NO3 (uM) | 0 |  | NO2/NO3 (uM) | 0 |
| **Total** | **532** |  | **Total** | **736** |

**S1 Table**
